# A molecular switch orchestrates enzyme specificity and secretory granule morphology

**DOI:** 10.1038/s41467-018-05978-9

**Published:** 2018-08-29

**Authors:** Suena Ji, Nadine L. Samara, Leslie Revoredo, Liping Zhang, Duy T. Tran, Kayla Muirhead, Lawrence A. Tabak, Kelly G. Ten Hagen

**Affiliations:** 1 Developmental Glycobiology Section, NIDCR, National Institutes of Health, 30 Convent Drive, Bethesda, MD 20892-4370 USA; 2 Section on Biological Chemistry, NIDCR, National Institutes of Health, 30 Convent Drive, Bethesda, MD 20892-4370 USA

## Abstract

Regulated secretion is an essential process where molecules destined for export are directed to membranous secretory granules, where they undergo packaging and maturation. Here, we identify a gene (*pgant9*) that influences the structure and shape of secretory granules within the *Drosophila* salivary gland. Loss of *pgant9*, which encodes an O-glycosyltransferase, results in secretory granules with an irregular, shard-like morphology, and altered glycosylation of cargo. Interestingly, *pgant9* undergoes a splicing event that acts as a molecular switch to alter the charge of a loop controlling access to the active site of the enzyme. The splice variant with the negatively charged loop glycosylates the positively charged secretory cargo and rescues secretory granule morphology. Our study highlights a mechanism for dictating substrate specificity within the O-glycosyltransferase enzyme family. Moreover, our in vitro and in vivo studies suggest that the glycosylation status of secretory cargo influences the morphology of maturing secretory granules.

## Abstract (web summary)

Regulated secretion is an essential process that requires packing and maturation of cargo proteins in membranous secretory granules. Here, authors identify a gene, *pgant9*, that influences the glycosylation status of the secretory cargo as well as the structure of secretory granules within the *Drosophila* salivary gland.

## Introduction

Regulated secretion is an essential process in many cells and tissues whereby bioactive molecules are synthesized and stored in membranous secretory vesicles until a trigger signals their release to the extracellular environment^1^. This type of secretion occurs across many organs, including the digestive, reproductive, endocrine, and nervous systems, and provides a means to deliver essential molecules in response to a hormonal or physiological stimulus^2,3^. Defects in the synthesis or appropriate secretion of the cargo can result in diverse diseases, such as diabetes, cystic fibrosis, and inflammatory bowel disorders^4–8^.

The biosynthesis of secreted molecules begins in the endoplasmic reticulum (ER), where proteins are synthesized, folded and then transported to the Golgi apparatus in a COPII-dependent process^9^. Within the Golgi apparatus, a variety of post-translational modifications occur before cargo is packaged into vesicles that bud from the trans-Golgi network (TGN). These immature secretory granules undergo a number of maturation steps including homotypic fusion; acidification of the granular lumen; and proteolytic processing and condensation of the secretory cargo^10–16^. Large, expanded, and highly-glycosylated secretory cargo such as mucins present unique structural challenges and are thought to undergo regulated condensation within the secretory granule as part of the maturation process. For example, the predominant mucin of the mammalian digestive tract, MUC2, undergoes pH and Ca^2+^-dependent multimerization and packaging^17^. However, the exact sequence of events and the many factors involved in cargo processing and proper granule maturation remain poorly understood. Likewise, the roles of protein modifications in cargo structure and condensation within the secretory granule remain to be investigated.

The *Drosophila* salivary gland synthesizes large secretory granules containing high molecular weight mucins that undergo hormone-regulated exocytosis prior to metamorphosis^18,19^. The size of the granules and the fluorescent tools developed by the *Drosophila* community, including fluorescently-labeled cargo proteins (Sgs3-GFP)^20^, allow one to image granule biogenesis and morphology in detail. Previous studies have shown that secretory granule biogenesis begins at the trans-Golgi network (TGN) where the highly glycosylated secretory cargo is loaded into small, immature granules in a clathrin and AP-1 dependent process^21^. Immature granules undergo a maturation process that remains ill-defined, but involves homotypic fusion^14,22^ that is regulated in part by type II phosphatidylinositol 4-kinase (PI4KII) and the SNARE Snap24^23^. Thus, as development proceeds, many small immature granules (1 μm diameter) fuse to form large, mature granules (3–8 μm diameter), which then await a hormone signal to begin the process of exocytosis. Upon hormone stimulation, mature secretory granules fuse with the apical plasma membrane and release their contents into the salivary gland lumen in a highly organized process that involves membrane mixing, fusion pore formation, and the recruitment of factors involved in linear and branched actin formation^24,25^. Granule collapse and extrusion of the viscous, highly-glycosylated cargo into the salivary gland lumen occurs in an actin and myosin-dependent manner^24–26^. While many factors involved in regulated secretion have been discovered using this system, the factors regulating granule morphology and maturation remain largely unknown.

Here, we use the *Drosophila* salivary gland to identify a gene (*pgant9*) affecting secretory granule morphology. Loss of *pgant9* results in secretory granules that adopt an irregular, shard-like appearance as they mature. Interestingly, *pgant9* encodes an O-glycosyltransferase that undergoes tissue-specific splicing within the salivary gland to orchestrate a change in enzyme specificity. The salivary gland-specific enzyme variant preferentially glycosylates the positively charged cargo proteins of the salivary gland and rescues granule morphology, suggesting that cargo glycosylation is essential for proper secretory granule morphology and maturation. Additionally, we identify a unique mechanism for altering enzyme specificity by solving the crystal structures of each splice variant. Taken together, our studies elucidate a factor that influences secretory granule morphology and provide evidence that the glycosylation status of cargo may influence granule shape. Moreover, we identify a strategy for modulating substrate specificity among members of this O-glycosyltransferase family.

## Results

### *pgant9* is required for proper secretory granule formation

**Fig. 1:**
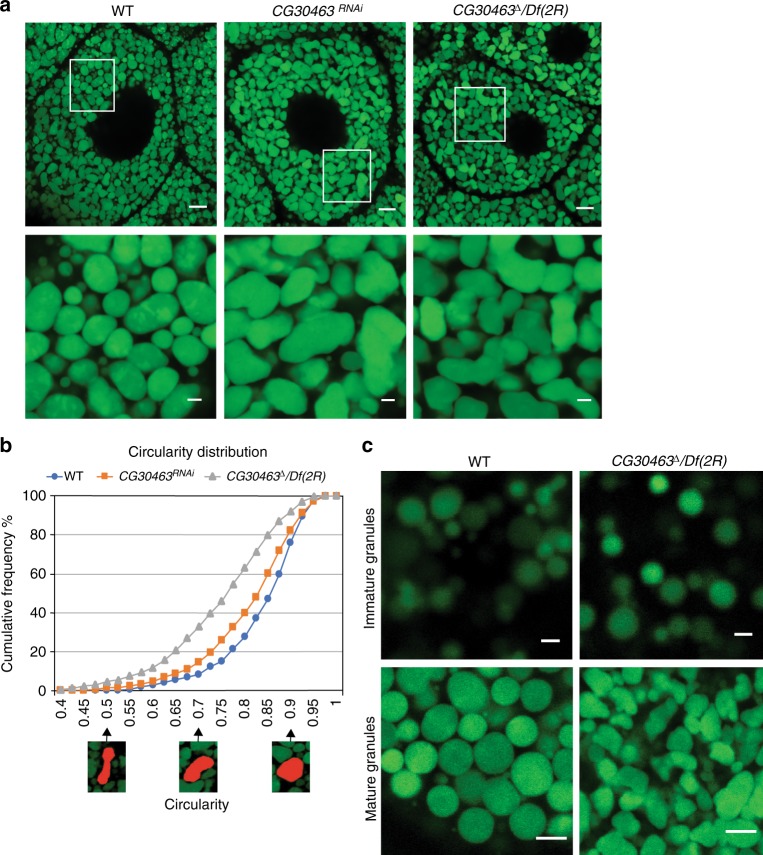
Irregular secretory granule morphology in the absence of *CG30463*. **a** Confocal images of third instar larval salivary glands (SGs) expressing Sgs3-GFP cargo (green) in secretory granules. Wild type secretory granules (WT*)* are round or oval in morphology while granules in SGs expressing RNAi to *CG30463* (*CG30463*^*RNAi*^) or deficient for *CG30463* (*CG30463*^*Δ*^*/Df(2R)*) are no longer round but have an angular, shard-like morphology. The white boxed area in the upper panels is enlarged below each image. Representative images from three independent experiments are shown. Scale bars, 10 μm for the upper panels and 2 μm for the lower panels. **b** Secretory granule circularity for WT, *CG30463*^*RNAi*^, and *CG30463*^*Δ*^*/Df(2R)* was calculated using the Nikon imaging software. Curves representing cumulative frequency of granule circularity for each genotype are shown. Images of secretory granules corresponding to circularity values of 0.5, 0.7, and 0.9 are shown below the *x*-axis. Granule circularity quantification was performed on granules from three individual salivary glands from each of three independent biological replicates of each genotype. Values were normalized by total number of granules (*n* = 815 for WT; *n* = 899 for *CG30463*^*RNAi*^; *n* = 841 for *CG30463*^*Δ*^*/Df(2R)*). **c** Confocal images of secretory granules expressing Sgs3-GFP in proximal (Immature Granules) or distal (Mature Granules) secretory cells of early third instar larval salivary glands from WT and in the absence of *CG30463* (*CG30463*^*Δ*^*/Df(2R))*. Scale bars, 1 μm for the upper panels and 5 μm for the lower panels. Representative images from three independent experiments are shown

Our laboratory has focused on the factors that influence regulated secretion using *Drosophila* secretory organs^14,24,27^. *Drosophila* lines carrying a fluorescently-labeled recombinant version of one of the major mucin proteins expressed in the salivary gland (Sgs3-GFP^20^), allow one to image secretory granule biogenesis and morphology^20,21,23–25^. Using this system, we find that loss of the conceptual gene *CG30463*, which was previously identified as a putative member of the UDP-GalNAc:polypeptide *N*-acetylgalactosaminyltransferase family of enzymes (ppGalNAcTs in mammals and PGANTs in *Drosophila*) responsible for the O-glycosylation of proteins^28–30^, influences the morphology of mature secretory granules (Fig. 1a). As shown in Fig. 1a, the parental (WT) Sgs3-GFP-containing secretory granules are round and relatively homogeneous in shape, while granules in salivary glands expressing RNAi to *CG30463* (*CG30463*^*RNAi*^) are highly irregular in size and shape, with an angular or shard-like appearance. Likewise, a deletion that removes exons 1–4 of the *CG30463* coding region over a deficiency that removes all of *CG30463* (*CG30463*^*Δ*^*/Df(2R)*; Supplementary Fig. 1) also resulted in secretory granules that were irregular in morphology (Fig. 1a). To quantitate differences in granule shape, we performed morphological analysis of circularity of the secretory granules in *WT*, *CG30463*^*RNAi*^ and *CG30463*^*Δ*^*/Df(2R)* using the Nikon NIS Elements imaging software suite as described in Methods using at least three biological replicates for each genotype. Average circularity values for each genotype are as follows: WT = 0.834 ± 0.0121; *CG30463*^*RNAi*^ = 0.806 ± 0.0215; and *CG30463*^*Δ*^*/Df(2R)* = 0.745 ± 0.0126. *P*-values for WT vs. *CG30463*^*RNAi*^ and WT vs. *CG30463*^*Δ*^*/Df(2R)* both were calculated to be <0.001. Cumulative frequency of circularity was graphed and is shown in Fig. 1b; a clear shift in the overall distribution of the circularity of secretory granules is seen upon knockdown (*CG30463*^*RNAi*^) or deletion (*CG30463*^*Δ*^*/Df(2R)*) of *CG30463* relative to WT, indicating that loss of *CG30463* results in aberrant secretory granule morphology.

To identify the stage at which granules adopted an irregular morphology, we next imaged early stages of secretory granule biogenesis. Interestingly, the defects in mature granule morphology seen upon loss of *CG30463* (*CG30463*^*Δ*^*/Df(2R))* were not present in the small, immature granules (<1 μm in diameter) emanating from the Golgi apparatus (Fig. 1c). These immature granules maintained a round, circular shape similar to that seen in WT. However, *CG30463*-deficient granules developed an irregular morphology at later stages of granule development (>2 μm in diameter) (Fig. 1c). This suggests that loss of *CG30463* is affecting some aspects of secretory granule maturation.

**Fig. 2:**
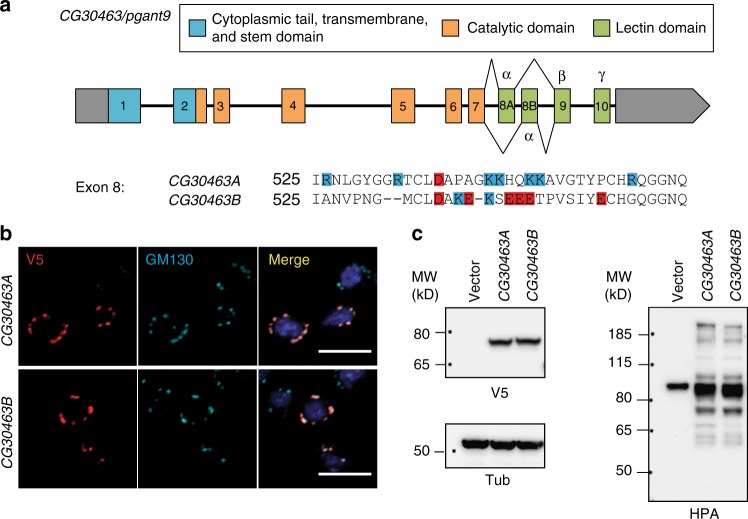
*CG30463/pgant9* encodes a Golgi-localized O-glycosyltransferase. **a** Gene structure for *CG30463/pgant9* is shown, with boxes representing exons and lines representing introns. The N-terminal (blue), catalytic (orange) and lectin (green) domains of the putative glycosyltransferase encoded by *CG30463* are shown. The lectin domain consists of three subdomains (α, β, and γ). The sequence for the differentially spliced α subdomain (exon 8) is shown, with acidic residues highlighted in red and basic residues highlighted in blue. **b** Both splice variants (V5-tagged; red) localized to the Golgi apparatus (as detected by anti-GM130; blue) in S2R+ cells. Scale bar, 10 μm. Representative images from two independent experiments are shown. **c** Western blots of S2R+ cells expressing vector alone (Vector), a V5-tagged recombinant *CG30463A* or a V5-tagged recombinant *CG30463B*. Panels on the left show *CG30463A* and *CG30463B* expression with the V5-tag (anti-V5) and loading controls (anti-tubulin). Panel on the right shows increased O-glycosylation (as detected by the lectin HPA) when *CG30463A* or *CG30463B* are expressed in S2R+ cells. Representative western blots from three independent experiments are shown. Molecular weight markers (kD) are shown to the left of each panel

To understand how the loss of *CG30463* is affecting secretory granule morphology, we next set out to characterize the protein encoded by *CG30463*. As mentioned previously, *CG30463* is predicted to encode a ppGalNAcT/PGANT, which is an O-glycosyltransferase responsible for the post-translational addition of the sugar *N-*acetylgalactosamine (GalNAc) to proteins, a modification typically found on mucins and other secreted and membrane-bound proteins. ppGalNAcTs are type II membrane proteins that belong to the CAZy family GT27^31^. These retaining glycosyltransferases, which catalyze the transfer of GalNAc from UDP-GalNAc to threonine or serine residues within protein substrates, are anchored to the Golgi membrane via a single pass transmembrane domain, and contain a luminal portion that consists of a stem region; a GT-A type catalytic domain with a Mn^2+^ ion coordinated by a DHH motif^32,33^; and a C-terminal lectin domain, which adopts a β-trefoil fold (belonging to the carbohydrate-binding module (CBM) group 13 in the CAZy database^31^). Interestingly, we found two distinct *Drosophila* cDNA clones emanating from the *CG30463* gene, differing only in a ~30 amino acid region (the α subunit) of the lectin domain (Fig. 2a). The lectin domain has been shown to bind extant GalNAc on previously glycosylated substrates to position the catalytic domain further away for additional GalNAc transfer^34,35^. Comparison of these clones to genomic sequence data revealed that they represent splice variants of exon 8 (Fig. 2a). This splicing event generates an α subunit with a net positive charge (isoform A, containing exon 8A) or a net negative charge (isoform B, containing exon 8B) (Fig. 2a). When expressed as full length proteins in *Drosophila* S2R+ cells, both isoforms localized to the Golgi apparatus (Fig. 2b) and generated an increase in O-glycosylation as detected by the lectin HPA (Helix pomatia agglutinin, which detects proteins modified with O-linked GalNAc) (Fig. 2c), suggesting that both encode functional, Golgi-localized O-glycosyltransferases. We hereafter refer to the gene *CG30463* as *pgant9* and designate the splice variants as *pgant9A* and *pgant9B* and the enzyme variants they encode as PGANT9A and PGANT9B.

### PGANT9A and B differentially glycosylate cargo proteins

**Fig. 3:**
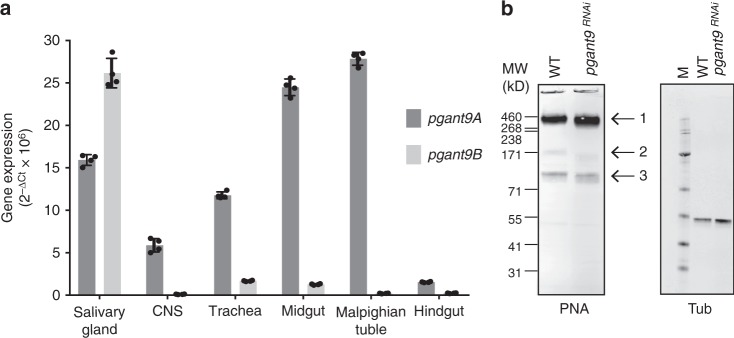
*pgant9* undergoes tissue-specific splicing. **a** Expression of each splice variant was quantitated in various larval tissues by qPCR. *pgant9B* expression is most abundant in the salivary gland while *pgant9A* is the predominant isoform in other tissues examined. RNA levels were normalized to 18S rRNA. Values represent mean ± s.d. from four experiments. **b** Western blots of salivary gland extracts from *WT* larvae or larvae expressing RNAi to *pgant9* (*pgant9*^*RNAi*^) probed with a lectin (peanut agglutinin; PNA) that detects the major salivary gland O-glycans. Size shifts in the three major PNA-reactive bands are seen (denoted with arrows on the right side of the panel). Tubulin loading control is shown in the lower panel. Representative western blot from four independent experiments are shown. Size markers are shown to the left of each panel

To investigate how *pgant9* splicing is regulated in vivo, we examined expression levels of each splice variant in larval tissues (Fig. 3a). *pgant9A* was the predominant form in most tissues (including the trachea, midgut, hindgut, CNS and Malpighian tubules) with the exception of the salivary gland. *pgant9B* was the predominant splice variant present in the larval salivary glands, suggesting a possible unique requirement for this isoform in that tissue.

To begin to identify the substrates of PGANT9A and PGANT9B in salivary glands, we performed western blotting and mass spectrometry on *WT* and *CG30463*^*RNAi*^ salivary glands. Westerns probed with a lectin (peanut agglutinin; PNA) that recognizes O-glycosylated proteins (Galβ1,3GalNAc-O-S/T)^36^ revealed mobility shifts in the three major PNA reactive bands upon knockdown of *pgant9* in salivary glands, suggesting changes in the O-glycosylation status of these proteins (Fig. 3b). Mass spectrometry identified one of the major components of bands 1 and 3 as the salivary gland mucin Sgs3 (Supplementary Tables 1 and 2). While band 3 likely represents a glycosylated version of an Sgs3 monomer or dimer, band 1 runs much higher than the predicted molecular weight of glycosylated Sgs3. This suggests that a population of Sgs3 exists as a high molecular weight multimer, possibly through bonding via cysteines present in the N-terminal and C-terminal regions, similar to what is seen for other mucins. The major component of band 2 was previously identified as the protein Eig71Ee (encoded by *CG7604*), another protein secreted from the salivary gland that has been shown to be O-glycosylated^37^. Interestingly, the repetitive/O-glycosylated regions of both Eig71Ee and Sgs3 are unique in that they contain positively charged amino acid residues amongst the threonine and serine residues predicted to be O-glycosylated.

**Fig. 4:**
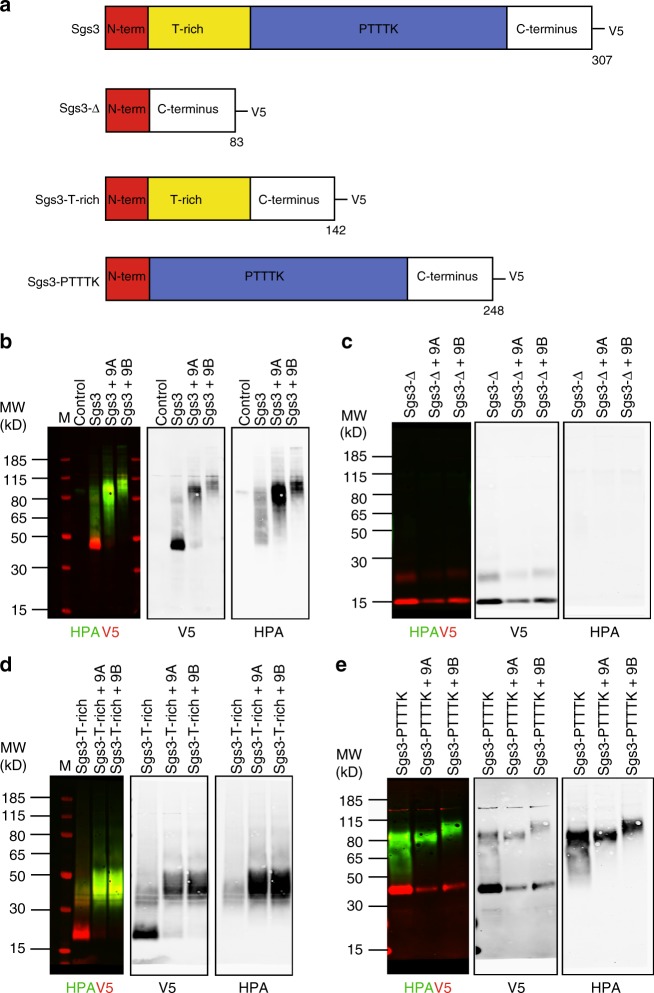
Differential glycosylation of Sgs3 by PGANT9A and PGANT9B. **a** Diagrams of full length Sgs3 and Sgs3 deletion constructs are shown (sequences are shown in Supplementary Fig. 2a). The N-terminal region is shown in red and the two distinct regions predicted to be glycosylated are shown in yellow (T-rich) and blue (PTTTK). The C-terminal region is shown in white. All constructs are V5-tagged. **b** Western blots of purified proteins from S2R+ cells transfected with empty vector (control), the full length Sgs3 construct (Sgs3), Sgs3 with a plasmid expressing PGANT9A (Sgs3 + 9A) or Sgs3 with a plasmid expressing PGANT9B (Sgs3 + 9B). Westerns were probed to detect the Sgs3 protein (V5; red) and changes in glycosylation (HPA; green). Westerns of purified proteins from similar transfections using the Sgs3-Δ construct (**c**), the Sgs3-T-rich construct (**d**), and the Sgs3-PTTTK construct (**e**) are also shown. Representative images from three independent experiments are shown. Size markers are shown to the left of each panel

To investigate whether Sgs3 is glycosylated by PGANT9A or PGANT9B, we co-expressed full length Sgs3 in *Drosophila* S2R+ cells with either PGANT9A or PGANT9B (Fig. 4 and Supplementary Fig. 2a). While both PGANT9A and PGANT9B were able to glycosylate Sgs3 (as detected by increased HPA reactivity and an increase in size), the size shift was reproducibly greater in the presence of PGANT9B, suggesting that PGANT9B confers increased glycosylation (Fig. 4b). Additionally, there did not appear to be an additive effect when both PGANT9A and PGANT9B were present together (Supplementary Fig. 2b). To investigate this further, we created deletion constructs of Sgs3 that eliminated one or both of the regions predicted to be glycosylated, and co-expressed them with either PGANT9A or PGANT9B. The first region contains stretches of threonines and is designated the threonine-rich (T-rich) region; the second region is comprised predominantly of repeating units of the sequence PTTTK and is designated the PTTTK region (Fig. 4a and Supplementary Fig. 2a). Deletion of both regions (Sgs3-Δ) resulted in no glycosylation by either PGANT9A or PGANT9B (Fig. 4c). Interestingly, while constructs containing only the T-rich region (Sgs3-T-rich) were glycosylated similarly (as shown by a similar size shift in the HPA-reactive material) in the presence of either PGANT9A or PGANT9B (Fig. 4d), constructs containing only the PTTTK region (Sgs3-PTTTK) showed a reproducible increase in the size of the HPA-reactive material in the presence of PGANT9B relative to PGANT9A, suggesting that this region is preferentially glycosylated by PGANT9B (Fig. 4e). Taken together, these data suggest that the T-rich region of Sgs3 is an in vivo substrate for both PGANT9A and PGANT9B, but PGANT9B preferentially acts on the positively-charged PTTTK repetitive region.

### Splicing of *pgant9* generates a unique charged loop

**Fig. 5:**
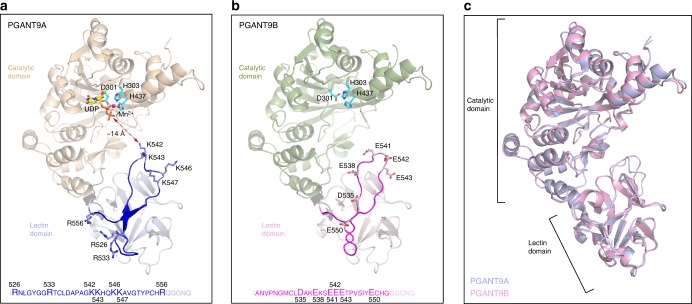
Crystal structures of PGANT9A and PGANT9B reveal divergent substrate preferences. **a** Crystal structure of PGANT9A with the catalytic domain shown in wheat and the lectin domain shown in light blue. The active site residues D301, H303, and H437 are in cyan, Mn^2+^ in magenta, and UDP in yellow. The variable α subunit is in dark blue, and the positively charged residues are shown as sticks. The variable region sequence is in dark blue and the residues shown in the structure are in larger font. The conserved residues on the peripheries are shown in light blue. **b** Crystal structure of PGANT9B with the catalytic domain in green, the lectin domain in pink, and the α subunit in magenta (with negatively charged residues shown as sticks). The variable region sequence is in magenta and the negatively charged residues are in larger font, while the conserved residues on the peripheries are in light pink. **c** PGANT9A and PGANT9B adopt the same overall fold. PGANT9A is in light blue, and PGANT9B is in pink

To understand how the lectin domain-α subunit influences enzymatic specificity, X-ray crystal structures of PGANT9A and PGANT9B in the presence of UDP, Mn^2+^, and an Sgs3 peptide substrate were solved at atomic resolution (Fig. 5; Supplementary Fig. 3; Table 1). PGANT9A and PGANT9B adopt the canonical ppGalNAcT tertiary structure where a ~10 amino acid flexible linker connects the N-terminal catalytic domain to the C-terminal lectin domain (Fig. 5a, b)^38–42^. PGANT9A crystallizes with 4 molecules in the asymmetric unit, and each molecule contains electron density in the active site consistent with a catalytic Mn^2+^ ion coordinated by the conserved active site residues Asp 301, His 303, and His 437, and 2 oxygens from the α and β phosphate of UDP (Fig. 5a). However, there is insufficient electron density to model in a peptide, and the partially ordered active site loop containing residues Arg 440 to Arg 448 is in a semi-closed conformation^43^. PGANT9B crystallizes with 2 molecules per asymmetric unit, but density for UDP, Mn^2+^, or a peptide is not present in the active site under these crystallization conditions. The active site residue Asp 301 is misaligned, and the active site loop is disordered (Fig. 5b).

**Table 1:** Data collection and refinement statistics

	PGANT9A^b^	PGANT9B^b^
	PDB 6E4Q	PDB 6E4R
*Data collection*		
Space group	*I* 1 2 1	*I* 1 2 1
*Cell dimensions*		
*a, b, c* (Å)	126.3, 168.8, 153.1	113.9, 48.4, 232.0
α, β, γ (°)	90, 106.3, 90	90, 91.4, 90
Resolution (Å)^a^	20.0–2.80 (2.90–2.80)	20.0–2.06 (2.14–2.06)
*R*_pim_^a^	0.125 (0.583)	0.081 (0.660)
*I/ σ*(*I*)^a^	6.00 (1.00)	8.64 (1.03)
Completeness (%)^a^	94.1 (70.0)	96.7 (83.4)
Redundancy^a^	6.8 (3.5)	3.2 (3.0)
*Refinement*		
Resolution	19.95–2.80	20.0–2.06
No. reflections	70,993	76,400
*R*_work_/*R*_free_	19.0/26.0	16.4/20.7
*No. atoms*		
Protein	16,242	8096
Mn^2+^	4	0
UDP/GlcNAc	100/56	0/28
Water/Solvent	194/36	711/86
*B-factors (Å*^*2*^*)*		
Average	53.6	37.2
Macromolecules	53.6	36.6
Solvent^c^	35.9	40.8
Ligands	67.9	57.5
*R.m.s deviations*		
Bond lengths (Å)	0.009	0.007
Bond angles (°)	1.16	0.99

^a^Values in parentheses are for highest-resolution shell

^b^Data from a single crystal were used for solving the structure

^c^Water, (poly)ethylene glycol, and glycerol

**Fig. 6:**
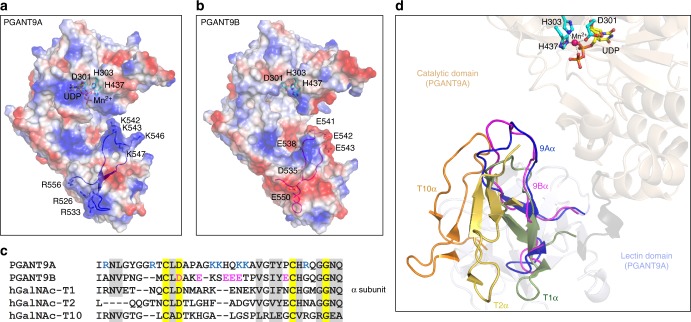
The α subunits of PGANT9A and PGANT9B determine their unique substrate specificities. **a** Electrostatic potential surface map of PGANT9A, where the blue regions are positively charged, and red regions are negatively charged superposed over the variable loop shown in dark blue. Basic residues in the loop are shown as sticks. **b** Electrostatic potential surface map of PGANT9B, where the blue regions are positively charged, and red regions are negatively charged superposed over the variable loop shown in magenta. Acidic residues in the loop are shown as sticks. **c** Sequence alignment of the α subunit of various isoforms. The α subunit is poorly conserved. Conserved residues are highlighted in yellow, and moderately conserved residues are highlighted in gray. The basic residues in PGANT9A are blue and the acidic residues in PGANT9B are in magenta. **d** The α subunits adopt distinct conformations. The α subunits of PGANT9A colored blue, PGANT9B colored magenta, ppGalNAc-T1 colored green, ppGalNAc-T2 colored yellow and ppGalNAc-T10 colored orange are superposed over the crystal structure of PGANT9A with the catalytic domain colored wheat, and the lectin domain colored light blue

PGANT9A and PGANT9B are superimposable, showing that differences in the α subunit between the 2 enzymes do not impact the orientation of the lectin domain relative to the catalytic domain and that the enzymatic specificity is exclusively sequence dependent (Fig. 5c). The α subunit, shown in dark blue in PGANT9A and magenta in PGANT9B, contains a charged loop (Fig. 5a, b, respectively). In PGANT9A, Lys 542, Lys 543, Lys 546, and Lys 547 in the α subunit loop form a basic patch that extends towards the active site with the shortest distance of ~14 Å from Lys 542 to the nearest UDP phosphate (Figs. 5a, 6a). Arg 526, Arg 533, and Arg 556 form a distinct basic patch at the opposite end of the α subunit (Figs. 5a, 6a). The distribution of positively charged residues in the α subunit of PGANT9A creates a predominantly positively charged patch that extends the entire length of the lectin domain (Figs. 5a, c, 6a), and the proximity of the positively charged loop to the active site suggests that it dictates preferences for substrates that contain negatively charged residues, as the positively charged surface could form non-specific electrostatic interactions with the substrate to position it for catalysis. Likewise, positively charged substrates might be excluded from accessing the active site given the position of the positively charged loop. The variable loop in PGANT9B adopts a similar conformation as the loop in PGANT9A (Fig. 5b, c), except that negatively charged residues Glu 538, Glu 541, Glu 542, and Glu 543 are clustered at the region of the loop near the active site to form a negatively charged patch (Figs. 5b, 6b). Another charged region is evident in the electrostatic potential map, where residues including Asp 535 and Glu 550 form a distinct negatively charged patch on the surface of the lectin domain (Figs. 5b, 6b).

Although the charged patches on the surface of the lectin domain consisting of Arg 526, Arg 533, and Arg 556 in PGANT9A and Asp 535 and Glu 550 in PGANT9B are distant from the active site, peptide substrates that are greater than ~16 amino acids can extend the entire length of the lectin domain as shown in the structure of ppGalNAc-T2 bound to Muc5AC^41^, supporting an additional role of the base of the α-subunit in potential substrate binding. Moreover, the peptides represent a fragment of what are usually much larger protein substrates that could potentially make contacts with various parts of the enzyme. Thus, both enzymes have two distinct charged patches in their lectin domain formed by the α-subunit that results in preferences to recruit either negatively (PGANT9A) or positively (PGANT9B) charged substrates to the active site for catalysis (Fig. 6a, b).

We wondered if the orientation of the α subunits in PGANT9A and PGANT9B were unique compared with other members of the ppGalNAcT family whose structures have been solved. While the α subunit is a highly variable region among ppGalNAcT isoforms (Fig. 6c), the overall fold of the α subunit is conserved. As shown by previous structures of ppGalNAc-T1, ppGalNAc-T2, and ppGalNAc-T10^38–41^, the orientation of the α subunit varies significantly among the isoforms (Fig. 6d). In PGANT9A and PGANT9B, the loop is closer to the active site than in the other ppGalNAcTs. In ppGalNAc-T1, which is the human homolog most like PGANT9, the α subunit is positioned further from the active site and the loop appears to be less extended (Fig. 6d). The α subunit of ppGalNAc-T10 also adopts a conformation similar to those of PGANT9A/B, but is shifted away from the active site when the catalytic domains of PGANT9A/B and ppGalNAc-T10 are aligned. Alignment of the catalytic domains of PGANT9A/B and ppGalNAc-T2 results in the misalignment of their lectin domains, where the ppGalNAc-T2 α subunit is positioned in the opposite orientation of the PGANT9A/B α subunit. As a result, the loop in the α subunit is furthest away from the active site in ppGalNAc-T2. Thus, the α subunit in PGANT9A/B is unique to both enzymes compared with the other isoforms in both its orientation and proximity to the active site. The region is also highly charged compared with other isoforms, which suggests that PGANT9 distinctively evolved to recognize highly charged substrates via its lectin domain and adopted splicing of the α subunit to alter that charge based on the needs of the cell/tissue.

### The charged loop controls peptide substrate specificity

**Fig. 7:**
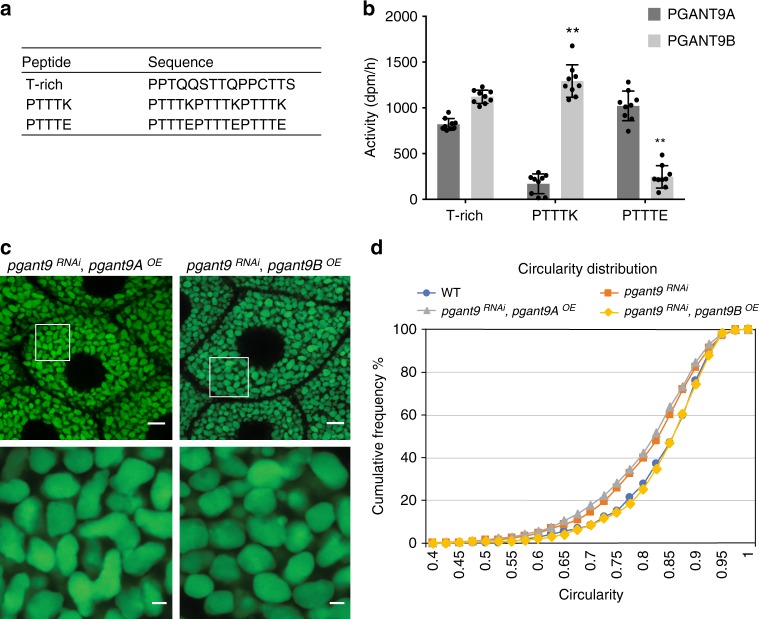
PGANT9B glycosylates Sgs3 and rescues secretory granule morphology. **a** Peptides used for in vitro reactions. T-rich is a peptide within the T-rich domain of Sgs3. PTTTK is a peptide within the positively-charged PTTTK repetitive region of Sgs3. PTTTE is a version of PTTTK where all positively-charged amino acids (K; lysine) have been replaced with negatively-charged amino acids (E; glutamic acid). **b** In vitro enzymatic assays measuring the glycosyltransferase activity of purified PGANT9A and PGANT9B against peptides derived from Sgs3. Values represent the mean ± s.d. from duplicate reactions. Assays were repeated three times. Student’s *t*-test was used to calculate *p* values (**p < 0.01). **c** Confocal images of third instar larval SGs from *pgant9*^*RNAi*^ larvae that overexpress either *pgant9A* (*pgant9*^*RNAi*^*, pgant9A*^*OE*^) or *pgant9B* (*pgant9*^*RNAi*^*, pgant9B*^*OE*^). All genotypes also express Sgs3-GFP (green). The white boxed area in the upper panels is enlarged below each image. Representative images from three independent experiments are shown. Scale bars, 10 μm for upper panels and 2 μm for lower panels. **d** Graphs of the cumulative frequency of granule circularity for each genotype are shown. Granule circularity quantification was performed on three individual SGs from three independent biological replicates. Cumulative frequency graph of *pgant9*^*RNAi*^*, pgant9A*^*OE*^ (*n* = 931 granules) and *pgant9*^*RNAi*^*, pgant9B*^*OE*^ (*n* = 946 granules) is overlapped with the data shown in Fig. 1b for WT and *CG30463*^*RNAi*^ (*pgant9*^*RNAi*^). QPCR data to verify mRNA expression of *pgant9A* and *pgant9B* are shown in Supplementary Fig. 4a and western blots to verify protein expression are shown in Supplementary Fig. 4b, c

To conclusively demonstrate the ability of the charged α subunit loop to dictate specificity toward charged substrates, we performed in vitro enzymatic assays with purified PGANT9A and PGANT9B, as described previously^29^. Putative substrate peptides were designed based on the predicted regions of glycosylation within Sgs3 (Fig. 7a). In agreement with what was seen in cell culture (Fig. 4), in vitro enzyme assays revealed that both PGANT9A and PGANT9B are capable of glycosylating the neutral T-rich peptide derived from the T-rich region (PPTQQSTTQPPCTTS), but only PGANT9B was able to efficiently glycosylate the positively charged PTTTK peptide derived from the PTTTK repetitive region (PTTTKPTTTKPTTTK) (Fig. 7b). To further interrogate this model, we redesigned the PTTTK peptide to replace all positively charged amino acids with negatively charged ones (PTTTE peptide; PTTTEPTTTEPTTTE) (Fig. 7a). As predicted, PGANT9A (with its positively charged α subunit loop) was able to efficiently glycosylate the negatively charged PTTTE peptide while PGANT9B displayed little activity (Fig. 7b). Taken together, our data support a model where tissue-specific splicing of the α subunit of the lectin domain creates a unique charged loop that influences access to the active site, thereby regulating the ability of PGANT9A and PGANT9B to modify highly charged substrates. This provides an example of both splicing altering the substrate specificity of a member of this enzyme family and changes within the lectin domain influencing preferences for unglycosylated substrates.

To address the isoform-specific roles of PGANT9A and PGANT9B in secretory granule morphology, we next performed rescue experiments with each individual isoform. Overexpression of each splice variant was verified at both the RNA and protein levels (Supplementary Fig. 4). Interestingly, overexpression of only *pgant9B* in salivary glands deficient for both isoforms (*pgant9*^*RNAi*^*, pgant9B*^*OE*^) resulted in the rescue of secretory granule morphology and circularity (Fig. 7c, d). Overexpression of *pgant9A* only (*pgant9*^*RNAi*^*, pgant9A*^*OE*^) did not rescue granule morphology/circularity (Fig. 7c, d). Average circularity values for each genotype are as follows: *pgant9*^*RNAi*^, *pgant9A*^*OE*^ = 0.798 ± 0.00555; *pgant9*^*RNAi*^*, pgant9B*^*OE*^ = 0.837 ± 0.0125. Taken together, our results provide evidence that differential splicing of an O-glycosyltransferase can alter substrate specificity by generating a charged loop that lies in proximity to the active site, regulating preferences for charged substrates. Moreover, our in vitro and in vivo studies suggest that the glycosylation status of secretory cargo influences the morphology of mature secretory granules.

## Discussion

Here we identify a factor that affects secretory granule morphology. Upon loss of *pgant9*, granules adopt an irregular shard-like appearance during maturation. Moreover, our in vitro and in vivo data indicate that the enzymatic product of *pgant9* specifically glycosylates the secretory cargo produced by the salivary gland, suggesting that defects in cargo glycosylation are influencing the maturation and final morphology of secretory granules. Glycosylation of proteins is known to influence their structure^44–46^. In particular, the high levels of O-glycosylation typically seen in the serine and threonine-rich repetitive regions of mucins are known to confer extended, rod-like structures to these regions^44,45,47^. MUC2, a highly O-glycosylated intestinal mucin, has been shown to dimerize via a C-terminal cysteine knot domain, and then trimerize via N-terminal von Willebrand domains in a pH and Ca^2+^-dependent manner^17,48,49^. The internal highly glycosylated mucin domains are thought to assemble into parallel rods during this process, allowing the N-terminal domains to interact and form the concatenated ring structures seen in secretory granules^17^. One can thus imagine how loss of glycosylation and collapse of the rod-like structure could interfere with the orderly packing and multimerization of other regions during granule maturation, thereby changing the structure of the packaged cargo and potentially influencing the shape of the secretory granule. A model where cargo glycosylation affects the packaging that occurs during granule maturation fits nicely with our data showing that early, immature secretory granules have a normal appearance but as granule fusion and maturation proceeds, abnormal morphologies develop. However, it remains possible that PGANT9B may have other substrates within the salivary gland that could also be contributing to changes in granule morphology. Ongoing studies are aimed at imaging secretory granules and their cargo via electron microscopy to determine how proteins are packaged as maturation proceeds in wild type granules and how that packaging may be altered in the absence of PGANT9B.

In this study, we also elucidate a mechanism that regulates enzyme specificity and substrate selectivity within the ppGalNAcT/PGANT family. From previous structural studies, we know that the ppGalNAcT enzymes consist of a catalytic domain and a tripartite lectin domain that are separated by a flexible linker region. Based on previous structural and biochemical studies, the lectin domain was thought to only recognize GalNAc sugars on previously glycosylated substrates, as illustrated in structures of ppGalNAc-T2 and ppGalNAc-T4 bound to glycosylated substrates, where the substrate GalNAc is bound to the lectin domain via a conserved sugar binding pocket in the α subunit^41,42^; this recognition then served to position the catalytic domain near unmodified residues so that additional GalNAcs can be added a certain distance away from the extant sugar^34,35,38,39,41,42^. No prior evidence existed for the lectin domain influencing the glycosylation of unglycosylated (unmodified) substrates. Indeed, previously solved crystal structures for members of this enzyme family show that the α subunit of the lectin domain is not close enough to the active site to be predicted to affect peptide recognition or access to the catalytic domain^38–42^ (Fig. 6). However, the structures presented herein show a unique α subunit loop that is longer and in closer proximity to the active site. Additionally, by solving the crystal structures of both splice variants, we provide the first evidence that splicing within the α subunit of the lectin domain serves as a molecular switch to change the charge of this extended loop, thus potentially modulating access to the active site within the catalytic domain. Moreover, we demonstrate in vitro that simply changing the charge of a peptide alters whether it is a preferred substrate for PGANT9A or PGANT9B, lending further support to a model where the charged α subunit modulates access of charged substrates to the active site of the enzyme. Many GTs contain substrate binding domains that are critical for their catalytic function^33,50^, but the lectin domain is unique to this family of glycosyltransferases. While the overall fold of the lectin domain is conserved, its orientation and primary sequence are variable throughout the family, resulting in a domain that fine tunes the substrate preference of each family member in a distinctive way, either by recognizing extant sugars on substrates or as we show in this study, by influencing peptide substrate preferences through electrostatic interactions. While our data support a model in which the charged lectin domain influences access of charged peptides to the active site of the catalytic domain, previous studies have suggested that certain lectin domains may also influence product release^35^. Future kinetic and binding studies will interrogate exactly how the unique lectin domains of PGANT9A and PGANT9B are influencing substrate specificity and catalytic efficiency. Regardless, the present study provides evidence that splicing can alter substrate specificity and that lectin domains have roles beyond GalNAc sugar recognition.

Our study also provides evidence that alternative splicing within *pgant9* allows appropriate glycosylation of substrates unique to the salivary gland. Indeed PGANT9B, which contains the negatively charged α subunit and prefers to act on positively charged substrates, is the predominant splice variant in the salivary gland. Interestingly, the major cargos of the salivary gland consist of the mucin-like proteins Sgs3 and Eig72Ee, both of which contain positively charged regions that are predicted to be glycosylated. Another major secreted mucin produced by the salivary gland is Sgs1^51^, which also contains positively charged regions that are predicted to be heavily glycosylated. This suggests that splicing within the α subdomain to generate PGANT9B may have evolved to ensure proper glycosylation of the positively charged substrates abundant within the salivary gland. Likewise, tissues that only express the PGANT9A variant (that prefers to act on negatively charged substrates) are those that have abundant expression of negatively charged mucins (e.g., *Mur18B* in the Malpighian tubules; http://flybase.org/reports/FBgn0030999). This study highlights a regulatory mechanism for modulating glycosyltransferase enzymatic specificity in vivo, according to the repertoire of substrates expressed in particular cells and tissues. Whether the expression and splicing of *pgants* is coordinated with substrate expression remains to be determined.

Whether other ppGalNAcT/PGANT family members are differentially spliced in vivo to generate alternative specificities remains to be investigated. While alternative splicing has been described for other ppGalNAcT family members^52,53^, no change in substrate specificity or sites of sugar addition were noted^53^. However, changes in substrate preferences may be challenging to detect in standard in vitro assays if the biologically relevant in vivo substrates are not known. Thus, many splicing events predicted from in silico analyses in other ppGalNAcT family members may confer unique specificities based on the tissues in which the splicing occurs and the predominant substrates expressed there. The additional degree of enzymatic variation conferred by splicing could make an already large family of enzymes (20 ppGalNAcTs in mammals and now 10 PGANTs in *Drosophila*)^29,52,54,55^ even more functionally diverse and may serve to fine-tune enzymatic specificities based on the needs of the cell/tissue.

The factors that affect secretion, including cargo synthesis, modification, packaging and delivery to the extracellular environment are biologically relevant as disruptions in secretion or aberrations in the synthesis or integrity of cargo can contribute to diverse human diseases^4–7,56,57^. For example, secreted mucins are abundant along epithelial surfaces such as the digestive and respiratory tracts, where they mediate hydration and form a gel-like layer that interacts with the microbiome and confers protection from infection and damage^5,46,58,59^. Many diseases of the digestive tract, such as colitis and colon cancer, have disruptions of the mucosal layer as one of their hallmarks^56,57,60^. Studies in model organisms have also highlighted a crucial role for the proper formation and secretion of this essential extracellular membrane^4,6,59,61,62^. As O-linked glycosylation is one of the modifications characteristic of this secreted membrane^46,52,55,60^, understanding how it is regulated at the enzymatic level as well as how the specific modifications influence protein structure, function, stability and/or bioavailability is essential. In this study, we demonstrate a regulatory paradigm for altering substrate selectivity among the enzymes that initiate O-glycosylation and demonstrate that altered O-glycosylation influences secretory granule morphology during maturation. Moreover, we demonstrate that expression of a particular splice variant (PGANT9B) that has specificity for the mucinous cargo of the salivary gland can restore secretory granule morphology, suggesting a key role for cargo glycosylation/structure in granule morphology. How this change in glycosylation specifically affects mucin structure during packaging and how it influences the biophysical properties of mucins once secreted will be the subject of future investigations. Moreover, understanding the role of this post-translational modification in secretory granule maturation and in mucin biology will aid in the development of mucin mimetics and strategies to treat diseases that are exacerbated by or the result of disruption of mucin biosynthesis and function.

## Methods

### *CG30463/pgant9* cloning and DNA constructs used

DNA encoding *pgant9* (*CG30463*) was amplified by PCR using cDNA made from embryonic total RNA (Clontech), forward (5′-GCGGAATTCGATATGGCCTTCATCTGGCGGCGAC-3′) and reverse (5′-CGCTCTAGACTCAACTTCGAGCTGTCGTAG-3′) primers, and was cloned into the *Eco*RI and *Xba*I sites of the pIB/V5-His vector (Invitrogen). All primers used for cloning and PCR are listed in Supplementary Table 3. The *pgant9A* isoform was distinguishable from *pgant9B* isoform by *Nco*I digestion. For expression of the secreted form of *pgant9A* and *pgant9B*, DNA fragments without the N-terminal cytoplasmic and transmembrane domains were amplified by PCR using pIB-*pgant9A* or *pgant9B* expression vector, forward (5′-CGCACGCGTACACGGATGACACCCGGC GT-3′) and reverse (5′-CTTGCGGCCGCTCACAACTTCGAGCTGTCGTAG-3′) primers, and then subcloned into *Mlu*I and *Not*I sites of pIMKF4^63^ to generate FLAG-tagged, secreted proteins. cDNA coding Sgs3 and Sgs3-PTTTK were synthesized by GenScript (Piscataway, NJ) and subcloned into the EcoRI and NotI sites of pIB-V5His vector. For Sgs3-∆ or Sgs3-T-Rich cloning, using pIB-Sgs3-V5His as template, PCR products were amplified by Sgs3-∆ forward (5′-TGGATGCCCCACCACACCTAAGCCGTG-3′) and reverse (5′-GTGGTGGGGCATCCACAATCGCAACAGTTG-3′); or Sgs3-T-Rich forward (5′-CACAACACCCACCACACCTAAGCCGTG-3′) and reverse (5′-GTGGTGGGTGTT GTGCACGGAAGTTGCG-3′) primers. With the PCR products, the In-Fusion HD EcoDry Cloning Kit (TaKaRa) was used to synthesize the Sgs3 deletions. For co-expression with Sgs3-V5 or Sgs3 deletions, V5-tag sequences of pIB-*pgant9A* and -*pgant9B* expression vectors were replaced with the FLAG Tag. Sequences of all plasmids were confirmed by DNA sequencing.

### Fly stocks and genetics

All fly stocks and crosses were kept on MM media (KD Medical, Inc.) at 25 °C. The deficiency line used was *Df(2R)Exel6064* (Bloomington #7546).  The *c135-Gal4*-driver line (Bloomington #6978) was used to generate RNAi in the salivary gland. To visualize glue granules, the *Sgs3-GFP* line (Bloomington #5885)^20^ was recombined with the *c135-Gal4*-driver line to generate *Sgs3-GFP, c135-Gal4*^24^. An RNAi target region for *pgant9* was designed using Snapdragon (http://www.flyrnai.org/snapdragon) and off-targets were screened using a 19 bp-size threshold. The C-terminal region of *pgant9* (300 bp) was selected and amplified with the sense primer (5′-CGACGACTCCTGTCTCGATT-3′) and the anti-sense primer (5′-GCTTGTGATTGTTGGTGTCG-3′) and cloned into pWIZ vector as described previously to generate the *UAS-pgant9*^*300IR*^ vector^30^. For overexpression of *pgant9A* or *pgant9B* in the fly, the pUAST vector was used to generate *UAS-pgant9A*^*OE*^ or *UAS-pgant9B*^*OE*^. DNA fragments were amplified by PCR using pIB-*pgant9A* or *pgant9B* expression vector, forward (5′-GCGGAATTCGATATGGCCTTCATCTGGCGGCGAC-3′) and reverse (5′- CTTGCGGCCGCTCACAACTTCGAGCTGTCGTAG-3′) primers, and then subcloned into *EcoR*I and *Not*I sites of pUAST vector. All transgenic lines were generated by BestGene (Chino Hills, CA). The *CG30463*^*Δ*^ deletion strain (where exons 1–4 of *CG30463* are deleted) was constructed through FRT- site-directed recombination using transposons *P{XP}d11168* (in the 5′UTR of *CG30463/pgant9*) and *P{Bac}CG30463*^*f03628*^ (in the 4th intron of *CG30463/pgant9*) through genetic crosses of these lines as described previously^64^. Potential deletion mutants were screened by loss of mini-white gene. Genomic DNA samples were obtained from these potential *CG30463/pgant9* mutants and used as templates for PCR amplification of a fragment encompassing the *CG30463/pgant9* locus (forward primer, 5′-GTCATTCACAGGCTGAGAACAAC-3′; reverse primer, 5′-CTTAAGCCCAGCTACTATCTCAGG-3′). As predicted by the genome location of the transposons, we verified that the recombination generated a deletion of 41,681 bp (2R:16,689,713 to 16,731,394) that exons 1–4 of *CG30463/pgant9. Oregon R* stock was used for qPCR assessing gene expressing in wild type tissues in Fig. 3a. For wild type control, *w*^*1118*^ (*VDRC#60000*) stock was crossed with *Sgs3-GFP, c135-Gal4* stock. The *Sgs3-GFP* line (Bloomington #5884)^20^ was used as the WT control in Fig. 1c. For rescue experiments, the *UAS-pgant9*^*RNAi*^ line (*UAS*-*pgant9* ^*300IR#1*^) was recombined with either *UAS-pgant9A*^*OE#Q2*^ or *UAS-pgant9B*^*OE#S8*^, and crossed to the *Sgs3-GFP, c135-Gal4* line. Fly crosses were performed on MM media (KD Medical) at 25 °C^30^.

### Salivary gland imaging and granule circularity measurements

Salivary glands from wandering third instar larvae were dissected in Schneider’s medium and transferred to glass bottom dishes (MatTek) containing 50 μl of media^24^. Samples were imaged on Nikon A1R+ confocal microscope with a Plan Apo IR X 60/1.27 numerical aperture (NA) water immersion (WI) objective. Imaging parameters for all samples such as wavelength, offset, detector, z-plane, zoom factor, and image resolution were kept constant. For granule circularity measurements, Nikon Imaging software (NIS-Elements AR version 4.5) was used to select individual granules that had clear outlines that were traceable by the software using the AutoDetect Binary tool. For analysis, 100 granules were selected by the AutoDetect. When the Autodetect outline aligned with the outer edge of the granule (within ~1–2 pixels), the granule was then added to the ROI (region of interest) list. After all of the granules were selected, the region of interest (ROI) was copied using the Binary toolbar and the measurement analysis tool was used to calculate the circularity of each selected granule. The software calculates circularity using the following formula: (4 × π × area/perimeter)^2^. A circularity value of 1 indicates a perfect circle while circularity values approaching 0 indicate an elongated polygon. Circularity analysis was performed on three independent biological replicates for each genotype. For each replicate, three glands were used for analysis and only three cells in each gland were used to measure a total of 800–950 individual granules per replicate. Values were exported to Microsoft Excel where the data was used to calculate frequency distributions for each genotype. The frequency bin was created with 0.5 intervals and data was normalized by dividing the frequency of the granules in the bin by the total number of granules analyzed. Values represent the mean ± s.d. *P*-values were calculated using a two-tailed Student’s *t*-test. No statistical method was used to predetermine sample size.

### Salivary gland dissection and Li-COR western blotting

PNA (Arachis hypogaea lectin, Sigma-Aldrich L0881) was labeled with IRDye 800CW (Licor 928–38044). Salivary glands from wandering third instar larva were dissected in PBS and transferred to a 1.5 ml eppendorf tube containing 50 μl of RIPA buffer (Sigma) containing 1× Halt Protease Inhibitor (Thermo Scientific). Protein extracts from 3 glands was loaded in each lane of a NuPAGE 4–12% Bis-Tris gel (Invitrogen). Gels were transferred onto nitrocellulose membranes. For Li-COR western blotting, the membranes were blocked with Odyssey Blocking Buffer (PBS-based) (Li-COR) and incubated with IRDye 800CW-conjugated PNA (1:5000) overnight at 4 °C. For the tubulin control blots, membranes were incubated with the tubulin antibody (1:1000) (Cell Signaling Technology, #2125) overnight at 4 °C, washed with PBS containing 0.1% Tween-20 (PBST) three times, and incubated with IRDye 680LT-conjugated anti-rabbit IgG (1:10,000) (Li-COR, #926–68021) for 1 h at room temperature. Membranes were washed with PBST three times, with PBS twice, and then scanned using a Li-COR Odyssey Infrared Imaging System. Full-length western blots are shown in Supplementary Fig. 5.

### S2R+ cell culture, immunostaining and western blotting

*Drosophila* S2R+ cells (*Drosophila* Genomics Resource Center, #150) were cultured in Schneider’s medium (Invitrogen) containing 10% heat-inactivated fetal bovine serum (Invitrogen) at 25 °C. For immunostaining, S2R+ cells were grown on cover glass and transfected with plasmids using Effectene transfection reagent (Qiagen) according to the manufacturer’s instructions. After 3 days, cells were fixed with 4% formaldehyde in phosphate buffered saline (PBS) and stained with the monoclonal Anti-V5-Cy3™ antibody (1:300) (Sigma, #V4014), the GM130 antibody (1:100) (Abcam, #ab30637), and Alexa Fluor® 647 AffiniPure F(ab’)_2_ Fragment Donkey Anti-Rabbit IgG (H + L) (1:200) (Jackson Immunoresearch, #711–606–152), each at room temperature for 1 h as described previously^65^. Samples were imaged on a Nikon A1R+ confocal microscope with a CFI Plan Apochromat Lambda X 60/1.4 numerical aperture (NA) oil immersion objective. For western blotting, S2R+ cells were grown on a 12-well plate and transfected with plasmids. After 3 days, cells were collected and lysed with RIPA buffer containing 1X Halt Protease Inhibitor (Thermo Scientific). Protein extracts were resolved using NuPAGE 4–12% Bis-Tris gels (Invitrogen) and transferred onto nitrocellulose membranes. For lectin blots, the membranes were blocked by 1X blocking buffer (Sigma, #B6429) and incubated with HRP-conjugated HPA lectin (1:1000) (EY Laboratories, Inc., #H-3601–1) diluted in blocking buffer overnight at 4 °C. After washing three times with PBS containing 0.1% Tween-20 (PBST), specific bands were visualized using enhanced chemiluminescence and analyzed by LAS 3000 Imaging System (Fujifilm). For immunoblotting, anti-V5-HRP (1:5000) (Invitrogen, #R961–25), anti-Tubulin (1:1000) (Cell Signaling Technology, #2125), and HRP-conjugated anti-rabbit antibody (1:2000) (Cell Signaling Technology, #7074) were used. For immunoprecipitation, 10 μl of agarose-conjugated V5 antibody (Sigma, #A7345) was incubated with cell lysates for 2 h at room temperature. Beads were washed with PBST four times and protein samples were eluted by LDS sample buffer (Invitrogen, #NP0007) containing β-mercaptoethanol. For Licor Western blotting, HPA (Helix pomatia lectin, Sigma-Aldrich L3382) were labeled with IRDye 800CW (Licor 928–38044). The membranes were blocked with Odyssey Blocking Buffer (PBS-based) (Li-COR) and incubated with IRDye 800CW-conjugated HPA (1:10,000) and anti-V5 antibody (1:5000) (Invitrogen, #R96025) overnight at 4 °C. Then the membranes were washed with PBS containing 0.1% Tween-20 (PBST) three times, and incubated with IRDye 680LT-conjugated anti-mouse IgG (1:10,000) (Li-COR, # 926–68020) for 1 h at room temperature. After wash, membranes were scanned using a Li-COR Odyssey Infrared Imaging System.The cell lines used tested negative to mycoplasma contamination.

### GalNAc transferase assays

Peptide substrates were synthesized by AnaSpec (Fremont, CA). Enzyme activity assays were performed as described previously^29^. Briefly, enzyme assays were performed using equal amounts of purified PGANT9A and PGANT9B. Each reaction was performed in triplicate at 37 °C for 1 h. Reactions were performed in 25 μl final volumes consisting of the following: 500 μM acceptor substrate, 7.3 μM ^14^C-UDP-GalNAc (54.7 mCi/mmol; 0.02 mCi/ml), 44 μM cold UDP-GalNAc, 10 mM MnCl_2_, 40 mM cacodylate (pH 6.5), 40 mM 2-mercaptoethanol and 0.1% Triton X-100. Reaction products were purified using anion exchange chromatography (AG 1 × -8, Bio-Rad). Reactions without acceptor peptide were used to generate background values that were subtracted from each experimental value. Adjusted experimental values for each substrate were then averaged and standard deviations were calculated. Enzyme activity (initial rate) is expressed as dpm/h.

### Quantitative RT-PCR

Quantitative RT-PCR (qPCR) was performed using the PCR primers designed using Beacon Designer software (BioRad) (*pgant9A* Sense: 5′-ATGGTATCTGGACAACATT-3′, Anti-sense: 5′-CTTCTTCTGGTGCTTCTT-3′; *pgant9B* Sense: 5′-AATGGTATCTGGACAACA-3′, Anti-sense: 5′-GGCACTCATAAATGGAAA-3′; 18S rRNA Sense: 5′- GGACACGCAAACTTCTCAACAGC-3′, Anti-sense: 5′-AATCTTCAGAGCCAATCCTTATCCC-3′). For tissue-specific expression of *pgant9* isoforms, third instar wandering larvae (Oregon R) were dissected in PBS and tissues were collected according to FlyAtlas guidelines (http://flyatlas.org/about_atlas.html). For RNAi efficiency and isoform specific overexpression, salivary glands from wandering third instar larvae were dissected in PBS and collected. RNA was isolated using RNAqeous Micro Total RNA kit (Invitrogen) and cDNA synthesis was performed using iScript cDNA Synthesis Kit (Bio-Rad). qPCR was performed on a CFX96 real time PCR thermocycler (Bio-Rad) using the SYBR-Green PCR Master Mix (Bio-Rad). RNA levels were normalized to 18S rRNA. Values represent the mean ± s.d. *P*-values were calculated using two-tailed Student’s *t*-test.

### Mass spectrometry

Salivary gland dissection, protein extraction and gel electrophoresis were carried out as described above. Gels were stained with Simply Blue Safe Stain (Invitrogen) using the protocol as suggested by the manufacturer. The three bands of interest were cut and sent for analysis to Poochon Scientific. The LC/MS/MS analysis was carried out using a Thermo Scientific Q-Exactive hybrid Quadrupole-Orbitrap Mass Spectrometer and a Thermo Dionex UltiMate 3000 RSLCnano System. Peptides from each sample were loaded onto a peptide trap cartridge at a flow rate of 5 μl/min. The trapped peptides were eluted onto a reversed-phase PicoFrit column (New Objective, Woburn, MA) using a linear gradient of acetonitrile (3–36%) in 0.1% formic acid. The elution duration was 60 min per fraction at a flow rate of 0.3 μl/min. Eluted peptides from the PicoFrit column were ionized and sprayed into the mass spectrometer, using a Nanospray Flex Ion Source ES071 (Thermo) under the following settings: spray voltage, 1.6 kV, Capillary temperature, 250 °C. Other settings were empirically determined. Raw data file was searched against public NCBI database of *Drosophila melanogaster* using the Proteome Discoverer 1.4 software (Thermo, San Jose, CA) based on the SEQUEST algorithm. Carbamidomethylation (+57.021 Da) of cysteines was fixed modification, and Oxidation and Deamidation Q/N-deamidated (+0.98402 Da), S/T O-GalNAc (+203.079 Da) were set as dynamic modifications. The minimum peptide length was specified to be five amino acids. The precursor mass tolerance was set to 15 ppm, whereas fragment mass tolerance was set to 0.05 Da. The maximum false peptide discovery rate was specified as 0.01. The resulting Proteome Discoverer Report contains all assembled proteins with peptides sequences and peptide spectrum match counts (PSM#).

### Protein preparation

PGANT9A (AA 146–650) and PGANT9B (AA 146–647) were cloned into the *Pichia pastoris* expression vector pPICZα A (Invitrogen) between the EcoRI and XbaI cut sites to generate His_6_-TEV-Gly_4_-PGANT9 constructs. The expression vectors were linearized with PmeI and transformed into SMD1168 (Invitrogen) cells by electroporation to generate stable PGANT9A and PGANT9B *P. pastoris* strains. Cells expressing PGANT9A or PGANT9B were grown in BMGY media, pH 6.0 in the presence of 100 μg/ml of Zeocin (Invivogen) at 30 °C to an OD_600_~10.0. To induce expression, cells were centrifuged at 1500×*g* for 15 min and resuspended in BMMY media, pH 6.0 in the presence of 100 μg/ml of Zeocin and grown at 20 °C for 24 hrs. The supernatant was collected by centrifugation at 1500×*g* for 15 min and further cleared by filtration, followed by the addition of 50 mM Tris pH 7.5 and 10 mM βME. All subsequent purification steps were carried out at 4 °C. The supernatant was loaded onto a 5 ml HisTrap HP (GE Healthcare) column equilibrated with buffer (A) containing 25 mM Tris, 250 mM NaCl, 10 mM βME, pH 7.5. The column was then washed with 10 Column Volumes (CV) of buffer A and the protein was eluted using a linear gradient of 30–300 mM imidazole over 10 CV. Peak fractions were pooled and dialyzed overnight in buffer A with 25 mM imidazole at 4 °C in the presence of 1.5 mg of His_6_-TEV protease to cleave the His_6_-Tag. The sample was passed through a 1 ml HisTrap HP (GE Healthcare) column to separate the pure PGANT9 from the His_6_-Tag and His_6_-TEV. Glycerol was then added to the tag-free PGANT9 to a final concentration of 20% and the protein was frozen in LN_2_ before storing at −80 °C.

High mannose chains were cleaved before preparing the samples for crystallization. Purified enzyme was first thawed and incubated with 50,000 units of MBP-tagged Endoglycosidase H (Endo Hf, New England Biolabs) per mg of enzyme and incubated overnight at 4 °C. The PGANT9 was separated from the Endo Hf on 2 ml of amylose resin (New England Biolabs) per mg of enzyme equilibrated with buffer (B) containing 100 mM NaCl, 20 mM HEPES, 0.5 mM EDTA, 10 mM βME, pH 7.3. The resin was washed with 2 CV of buffer B and the flow-through and wash containing PGANT9 were pooled. The protein sample was concentrated in a 30,000 kDa cutoff centrifugal filter (Amicon, Millipore) to 100 μl and buffer exchanged by adding 4 ml of buffer B and concentrating to 100 μl. To obtain the final sample for crystallization studies, 4 ml of buffer B were added a second time, and the protein was concentrated to 10 mg/ml. The activity of the *P. pastoris* purified enzymes against the Sgs3 substrates was verified as described in above.

### Crystallization, data collection and structure refinement

The PGANT9-peptide-UDP-Mn^2+^ complex was prepared by combining PGANT9A or PGANT9B with 5 mM peptide substrate (PTTTE for PGANT9A and PTTTK for PGANT9B (Anaspec, Fremont, CA), see Fig. 7a), 5 mM UDP, and 5 mM MnCl_2_ to a final protein concentration of 6 mg/ml, and the complex was incubated at RT for 30 min before placing on ice. Crystallization was initiated by mixing 1 μl of complex with 1 μl of well buffer using the hanging drop vapor diffusion method. PGANT9A crystals grew as 200 μM × 200 μM square-shaped plates in 22% PEG3350, 0.2 M sodium malonate, and 20 mM strontium chloride hexahydrate. PGANT9B crystals grew as 300 μM × 50 μM rods in 20% PEG3350 and 0.18 M ammonium citrate. Crystals of PGANT9A and PGANT9B were cryo-protected with well buffer containing 20% glycerol and flash frozen in liquid nitrogen. X-ray data was collected at the Advanced Photon Source using the SER-CAT BM- and ID-22 beam lines (Argonne, IL) 360 degrees of data were collected for PGANT9A and 300 degrees of data were collected for PGANT9B. Both data sets were collected at the wavelength of 1.0 Å and processed and scaled using HKL2000^66^. The structure of PGANT9B was solved by molecular replacement using MolRep (CCP4i)^67,68^ and human GalNAc-T1 as a search model (PDB: 1XHB). The lectin domain was manually rebuilt in Coot^69^ and the structure of PGANT9B was used for solving the structure of PGANT9A by molecular replacement (MolRep, CCP4i). The structures of PGANT9A and PGANT9B were refined in PHENIX^70^ to 2.8 and 2.07 Å resolution, respectively (Table 1). For PGANT9A, 97% of residues are in the favored region of the Ramachandran plot, 2.6% are allowed, and 0.15% are outliers. For PGANT9B, 97.3% of residues are in the favored region of the Ramachandran plot, 2.7% are allowed, and 0% are outliers. Figures and electrostatic potential maps were generated in Pymol (The PyMOL Molecular Graphics System, Version 2.0 Schrodinger, LLC).

### Statistical analyses

Number of replicates used for each analysis is specified in the figure legends. For granule morphology, every granule analyzed was used. *P*-values were calculated using the two-tailed Student’s *t*-test. No statistical method was used to predetermine sample size, no randomization methods were used for these studies and no blinding studies were performed.

### Data availability

Data supporting the findings of this manuscript are available from the corresponding author upon reasonable request. The coordinates and structure factors have been deposited in the Protein Data Bank under the accession codes: PDB 6E4Q (PGANT9A) and PDB 6E4R (PGANT9B). The mass spectrometry proteomics data have been deposited to the ProteomeXchange Consortium via the PRIDE partner repository with accession numbers PXD010548 (band 1) and PDX010549 (band 3).

## Electronic supplementary material

Supplementary Information
