# Supplementary material for: A molecular switch orchestrates enzyme specificity and secretory granule morphology
Source: Nat Commun. 2018 Aug 29;9:3508. doi: 10.1038/s41467-018-05978-9 (PMC6115407; doi:10.1038/s41467-018-05978-9)
Supplement: Supplementary file 1 — Supplementary Information [file 41467_2018_5978_MOESM1_ESM.pdf]

Supplementary Information for

A MOLECULAR SWITCH ORCHESTRATES ENZYME SPECIFICITY AND SECRETORY GRANULE MORPHOLOGY

Ji and Samara et al.

Supplementary Figures 1-5
Supplementary Tables 1-3
Supplementary Methods

Supplementary Figure 1

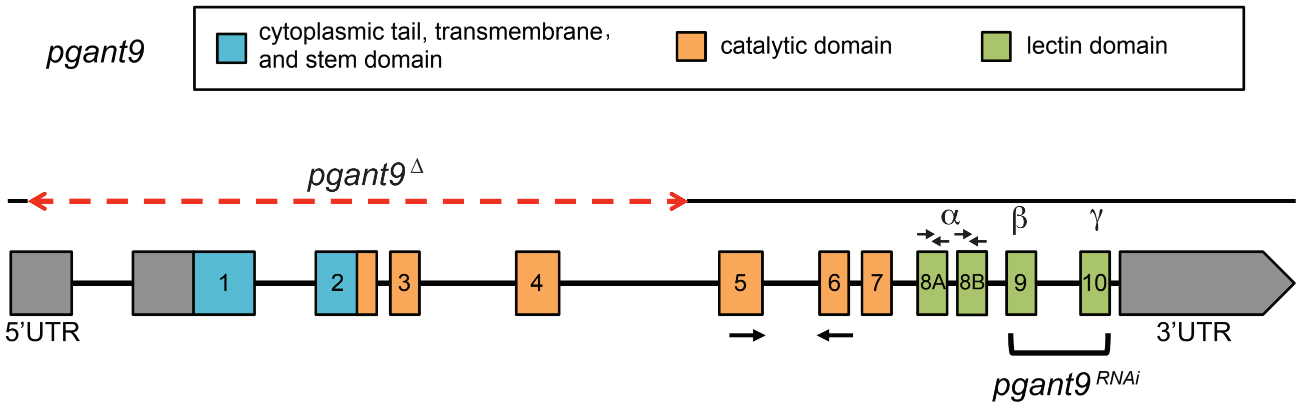

Supplementary Figure 1. Deletion constructs for *pgant9*^{RNAi} and *pgant9*$^{\Delta}$. The gene structure for *CG30463/pgant9* is shown as in Figure 2a. The region used to target *pgant9* by RNAi occurs in exons 9-10 (indicated by the black bracket) while the *pgant9* deletion (*pgant9*$^{\Delta}$) spans exons 1-4 (indicated by the red dotted arrow). The small black arrows indicate the regions that were used to generate PCR primers.

Supplementary Figure 2

a

Sgs3 (307 AA) (32kDa)
MELTIATALASILLIGSANVANCCDCGCPTTTTTCAPRTTQPPCTTTTTTTTTTC
APPTQQSTTQPPCTTSKPTTPKQTTTQLPCTTPTTTKATTTKPTTTKATTTKA
TTTKPTTTKQTTTQLPCTTPTTTKQTTTQLPCTTPTTTKPTTTKPTTTKPTTTK
PTTTKPTTTKPTTTKPTTTKPTTTKPTTTKPTTTKPTTTKPTTTKPTTTKPTTTK
PTTTKPTTTKPTTTKPTTTKPTTTKPTTTKPTTTKPTTPKPCGCKSCGPGGEP
CNGCAKRDALCQDLNGVLRNLERKIRQCVCGEPQWLL

Sgs3-T-rich (142 AA) (15kDa)
MKLTIATALASILLIGSANVANCCDCGCPTTTTTCAPRTTQPPCTTTTTTTTTTC
APPTQQSTTQPPCTTSKPTTPKQTTTQLPCTTPTTPKPCGCKSCGPGGEPC
NGCAKRDALCQDLNGVLRNLERKIRQCVCGEPQWLL

Sgs3-Δ (83 AA) (9kDa)
MKLTIATALASILLIGSANVANCCDCGCPTTPKPCGCKSCGPGGEPCNGCAK
RDALCQDLNGVLRNLERKIRQCVCGEPQWLL

Sgs3-PTTTK (248 AA) (26kDa)
MKLTIATALASILLIGSANVANCCDCGCPTTTKATTTKPTTTKATTTKATTTKPT
TTKQTTTQLPCTTPTTTKQTTTQLPCTTPTTTKPTTTKPTTTKPTTTKPTTTKP
TTTKPTTTKPTTTKPTTTKPTTTKPTTTKPTTTKPTTTKPTTTKPTTTKPTTTKP
TTTKPTTTKPTTTKPTTTKPTTTKPTTTKPTTPKPCGCKSCGPGGEPCNGCA
KRDALCQDLNGVLRNLERKIRQCVCGEPQWLL

b

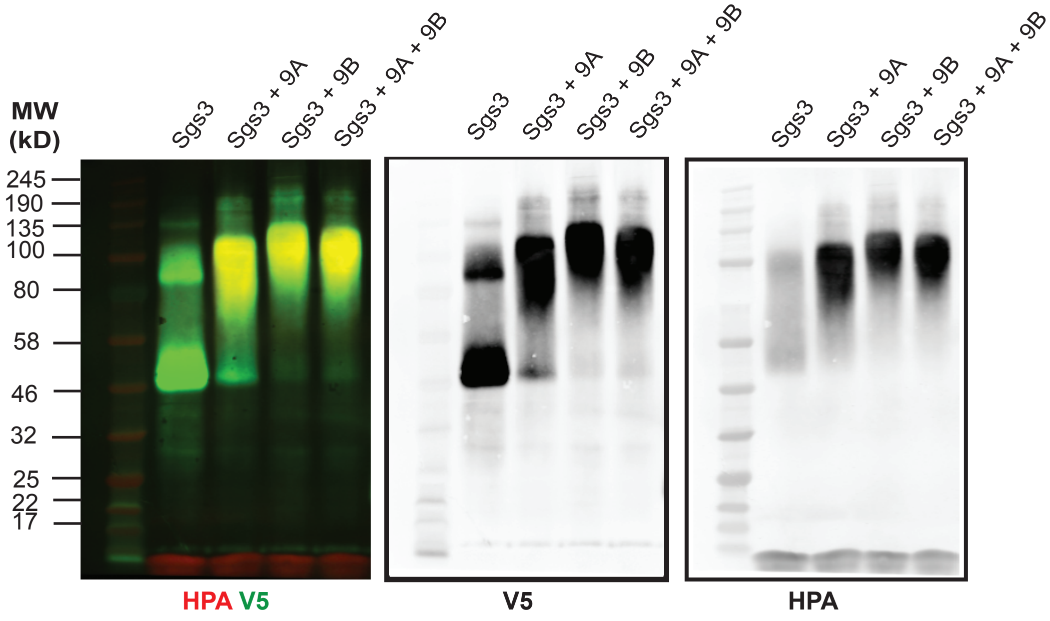

Supplementary Figure 2. Sequences of Sgs3 constructs. (a) Amino acid sequences of the different Sgs3 constructs used in Figure 4. Different protein regions are color coded as follows: N-terminus is shown in red, T-rich region is shown in yellow, PTTTK region is shown in blue and the C-terminus is shown in green. (b) Co-expression of PGANT9A and/or PGANT9B with full length Sgs3 (V5 tagged). Western blots were probed with V5 antibody and HPA.

Supplementary Figure 3

a

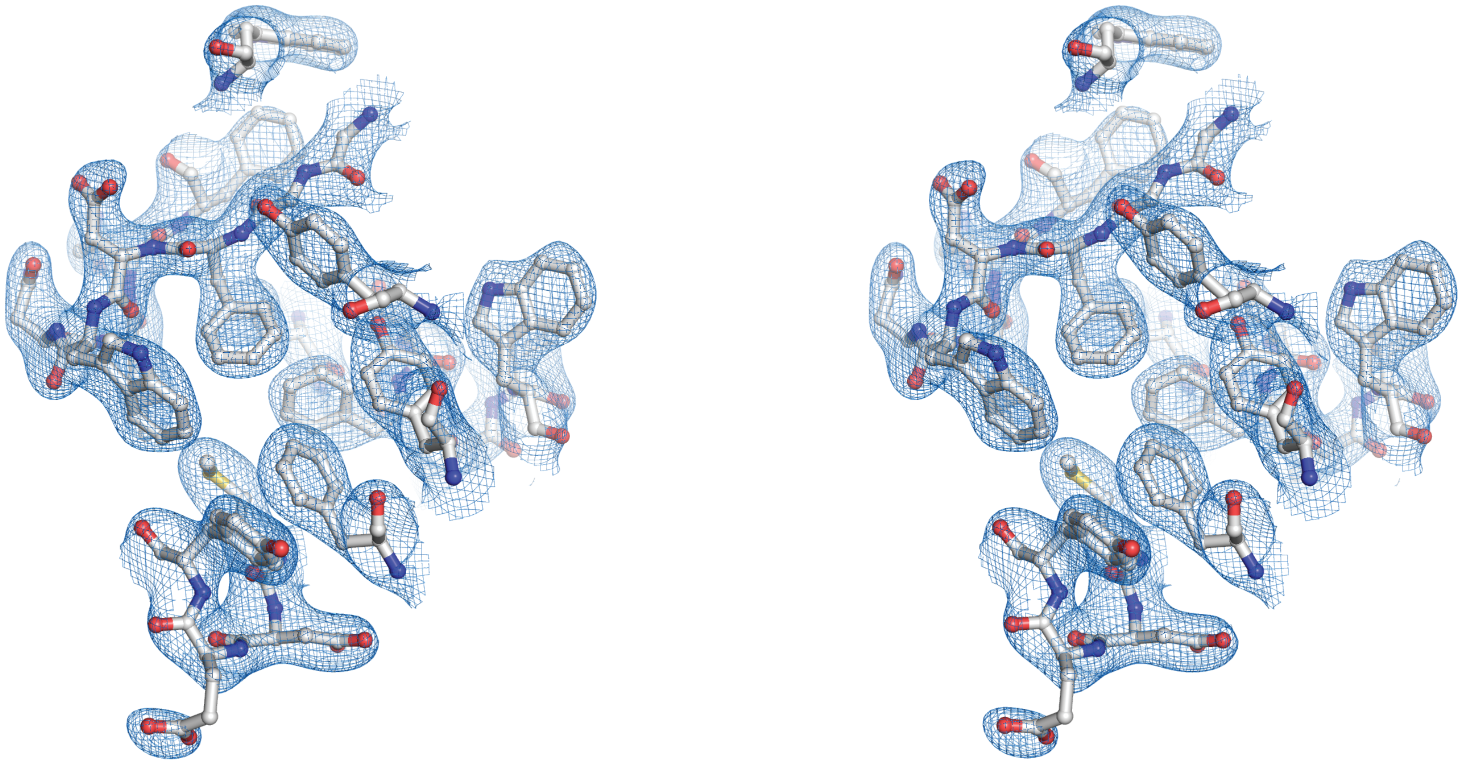

b

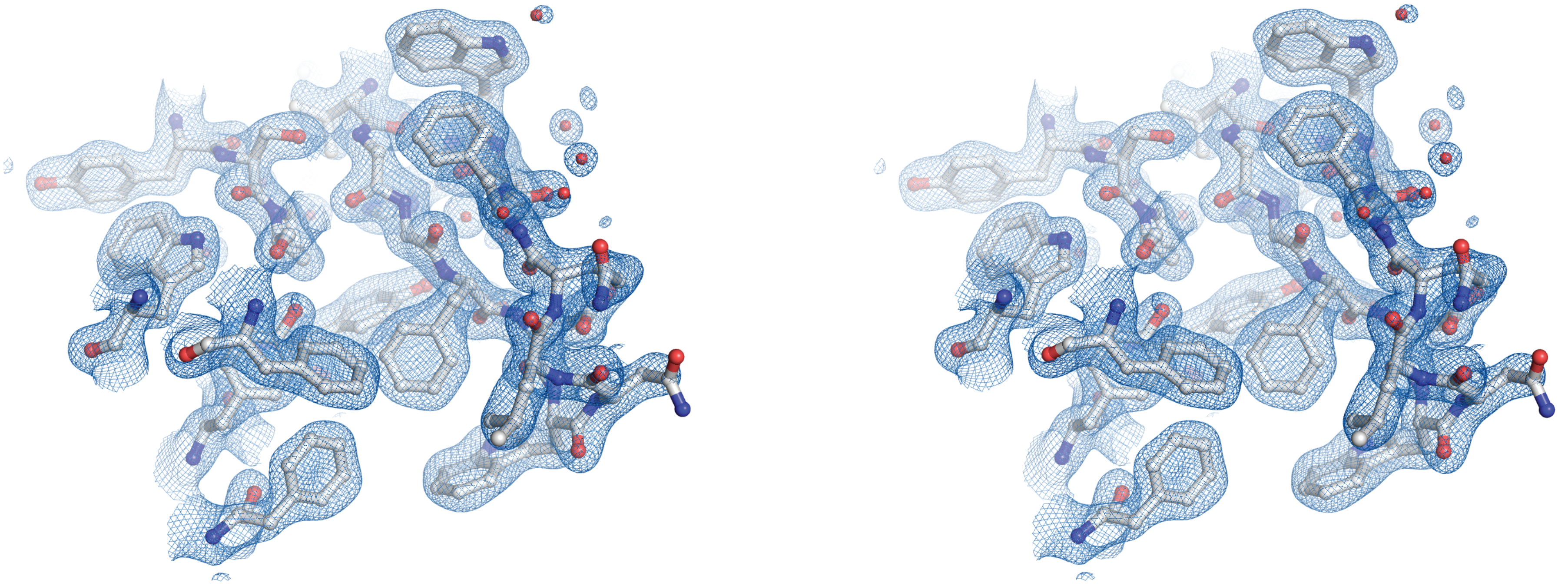

Supplementary Figure 3. Electron density maps for sample regions in PGANT9A and PGANT9B shown in stereo.
(a) 2.80 Å resolution 2Fo-Fc map (blue) contoured at 1.2σ superposed over a region in the catalytic domain of PGANT9A.
(a) 2.06 Å resolution 2Fo-Fc map (blue) contoured at 1.2σ superposed over a region in the catalytic domain of PGANT9B.

Supplementary Figure 4

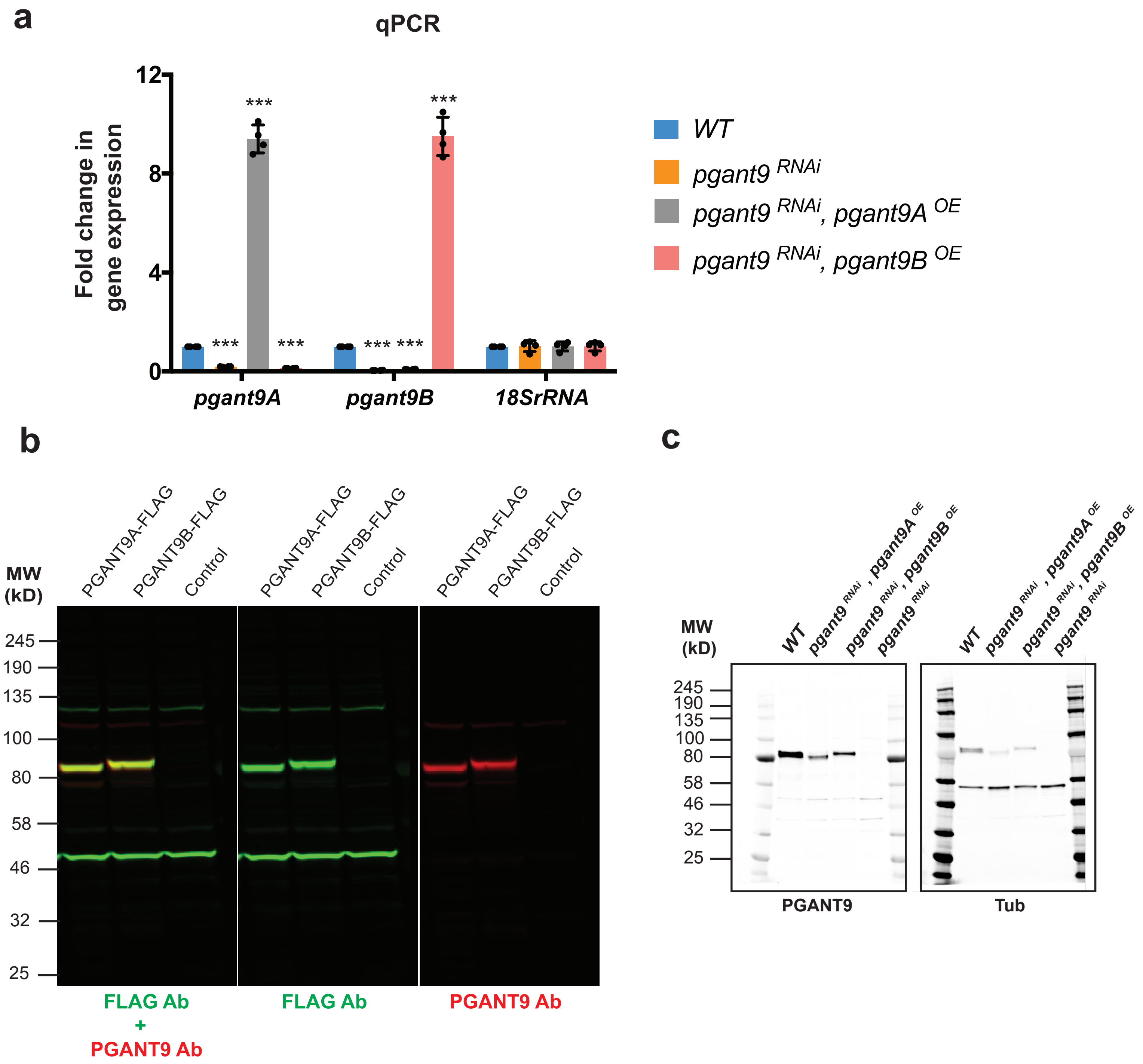

Supplementary Figure 4. Verification of PGANT9A and PGANT9B expression.
(a) qPCR to quantitate transcript levels of *pgant9A* and *pgant9B* after overexpression and in the *pgant9* ^{RNAi} knockdown condition. RNA levels were normalized to 18SrRNA. Values represent the mean ± s.d. from four experiments. Student's t-test (comparing each with *WT)* was used to calculate *P*-values. ***P<0.001. (b) Antibodies to the region common to PGANT9A and PGANT9B were raised and tested for specificity in cell culture. Western blots of recombinant PGANT9A-FLAG, PGANT9B-FLAG or vector alone (control) expressed in S2R+ cells were probed with anti-PGANT9 (PGANT9 Ab) or anti-FLAG (FLAG Ab). (c) Westerns of extracts from salivary glands of *WT*; *pgant9* ^{RNAi}, *pgant9A* ^{OE}; *pgant9* ^{RNAi}, *pgant9B* ^{OE}*;* or *pgant9* ^{RNAi} animals were probed with the anti-PGANT9 antibody to demonstrate the presence or absence of PGANT9 protein. Tubulin loading control is shown in the right panel.

Supplementary Figure 5

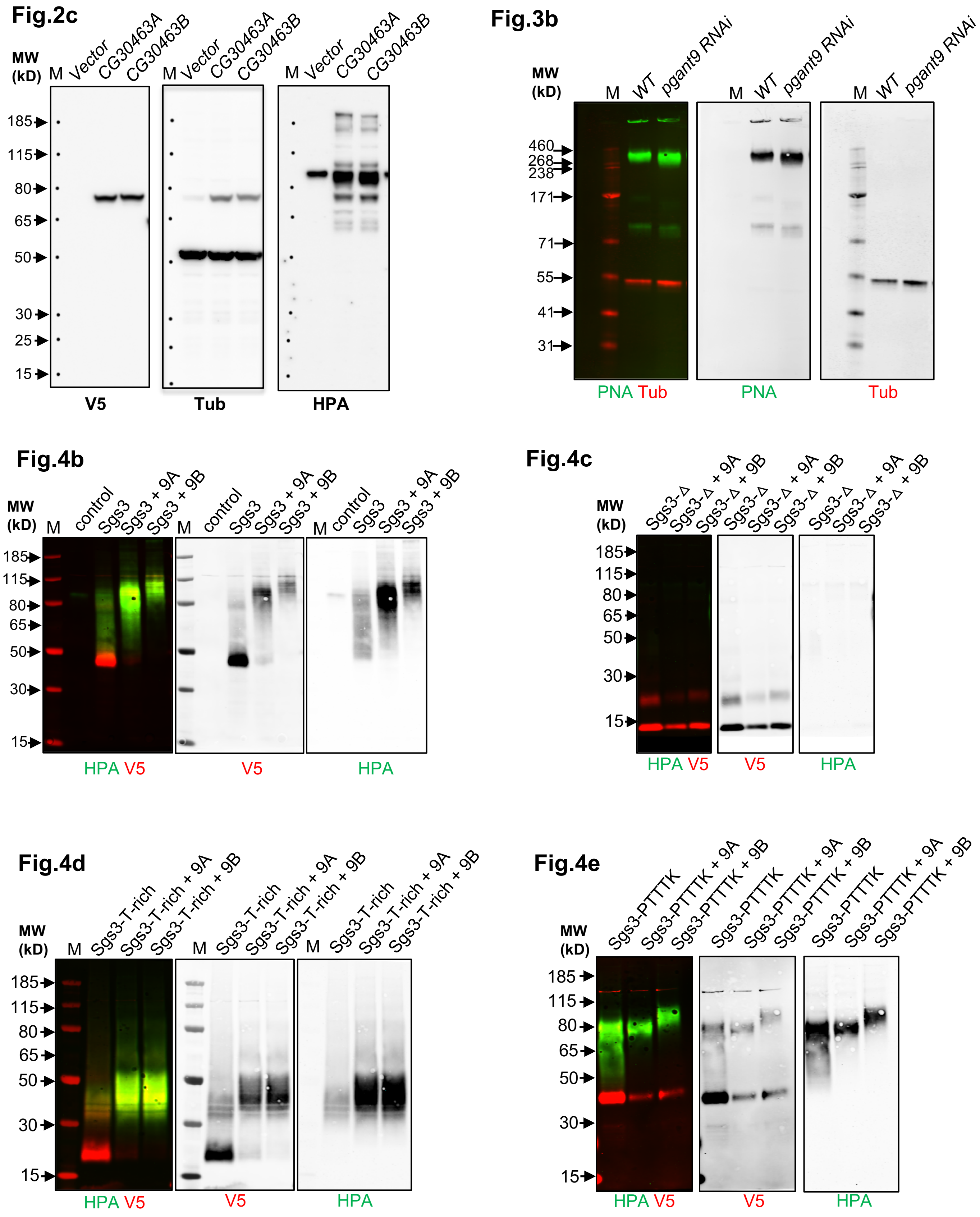

Supplementary Figure 5. Full-length western blots. Full-length westerns for each corresponding figure are shown.

Supplementary Table 1. Mass spectrometry results for the proteins identified in band 1. Sgs3 is highlighted in red.

Accession	Description	Score	Coverage	# Proteins	# Unique Peptides	# Peptides	# PSMs	# AAs	MW [kDa]	calc. pI
134467	**Salivary glue protein Sgs-3; Flags: Precursor**	**388.95**	**14.66**	**1**	**9**	**9**	**127**	**307**	**32.2**	**9.89**
12644115	Actin-5C; Flags: Precursor	164.38	49.47	4	7	10	72	376	41.8	5.48
51338816	Myosin heavy chain, non-muscle; AltName: Full=My	41.32	7.92	1	13	13	17	2057	236.5	5.68
25091355	Tubulin beta-1 chain; AltName: Full=Beta-1-tubulin	35.22	19.02	1	1	3	14	447	50.1	4.86
48429158	Tubulin beta-2 chain; AltName: Full=Beta-2-tubulin	31.25	18.39	1	1	3	14	446	49.8	4.83
13124683	Actin, larval muscle; AltName: Full=Actin-79B; Flag	23.19	19.68	5	1	4	7	376	41.8	5.48
135396	Tubulin alpha-1 chain	21.42	13.56	2	2	3	11	450	49.9	5.14
17380352	Elongation factor 2; Short=EF-2	16.67	2.84	1	1	1	6	844	94.4	6.60
50402125	Histone H4	12.71	33.98	1	4	4	5	103	11.4	11.36
46397771	Histone H3	7.92	23.53	1	1	1	4	136	15.4	11.27
134470	Salivary glue protein Sgs-5; Flags: Precursor	5.73	14.72	1	2	2	2	163	18.8	7.72
74868669	Filamin-A; Short=FLN-A; AltName: Full=Actin-bin	5.16	3.17	1	4	4	4	2210	239.0	6.11
47117002	Polycomb protein Pcl; AltName: Full=Polycomblike	3.67	1.63	1	1	1	3	1043	114.6	8.85
266311	Heat shock 70 kDa protein cognate 4; AltName: Full	3.04	2.61	2	1	1	1	651	71.1	5.52
10720262	Small nuclear ribonucleoprotein-associated protein B	2.64	11.06	1	1	1	1	199	21.0	11.22
73920809	Tubulin beta-3 chain; AltName: Full=Beta-3-tubulin	2.60	8.59	1	1	1	1	454	50.8	4.88
134471	Salivary glue protein Sgs-7; Flags: Precursor	2.54	14.86	1	1	1	1	74	7.9	7.83
91208165	Serine/threonine-protein kinase Smg1	2.51	0.56	1	1	1	1	3218	361.4	6.86
110831810	Zinc finger protein hangover	2.21	0.68	1	1	1	1	1913	209.6	5.30
55977067	Histone H3.3; AltName: Full=H3.3Q; AltName: Full	2.13	23.53	1	1	1	1	136	15.3	11.27
32130434	Alpha-mannosidase 2; AltName: Full=Golgi alpha-m	2.02	0.90	1	1	1	1	1108	126.6	7.20
135410	Tubulin alpha-2 chain	2.00	9.13	1	1	2	2	449	49.9	5.20
6685998	Basic helix-loop-helix neural transcription factor TA	1.77	2.51	1	1	1	1	398	44.8	7.01
56405335	Dynein heavy chain, cytoplasmic; AltName: Full=Dy	0.00	0.50	1	1	1	1	4639	529.9	6.33
126746	205 kDa microtubule-associated protein	0.00	2.95	1	1	1	1	1185	126.6	4.86
122054	Histone H2B	0.00	12.20	2	1	1	1	123	13.7	10.35
5921205	ATP synthase subunit alpha, mitochondrial; AltName	0.00	3.62	1	1	1	1	552	59.4	9.01
14285645	Odorant receptor 19a	0.00	9.82	2	1	1	1	387	44.2	9.17
2500630	DNA-directed RNA polymerases I, II, and III subuni	0.00	19.85	1	1	1	1	131	14.7	4.34

Supplementary Table 2. Mass spectrometry results for the proteins identified in band 3. Sgs3 is highlighted in red.

Accession	Description	Score	Coverage	# Proteins	# Unique Peptides	# Peptides	# PSMs	# AAs	MW [kDa]	calc. pI
113258	Actin-87E; Flags: Precursor	21.45	31.65	2	1	7	10	376	41.8	5.48
119142	Elongation factor 1-alpha; Short=EF-1-alpha	2.21	2.39	3	1	1	1	461	50.5	9.09
119881	Fasciclin-1; AltName: Full=Fasciclin I; Short=FAS I	2.13	4.14	1	2	2	2	652	72.6	7.02
121953117	Eukaryotic translation initiation factor 3 subunit B; S	12.65	10.29	1	9	9	10	690	80.4	6.28
122087256	Cysteine--tRNA ligase, cytoplasmic; AltName: Full=	6.95	8.23	1	6	6	6	741	84.2	6.30
122102832	Probable protein phosphatase CG10417	3.90	10.42	1	4	4	4	662	72.3	4.58
123661	Heat shock protein 83; AltName: Full=HSP 82	159.07	48.95	1	46	46	79	717	81.8	5.02
12644023	Protein hu-li tai shao; AltName: Full=Adducin-like p	2.59	2.16	1	1	1	1	1156	127.9	6.39
12644024	Protein ROP	0.00	6.87	1	1	1	1	597	67.8	6.70
12644115	Actin-5C; Flags: Precursor	72.91	60.90	3	7	13	35	376	41.8	5.48
12644120	Larval serum protein 1 beta chain; AltName: Full=H	190.74	37.90	1	38	40	95	789	95.8	5.64
13124701	Broad-complex core protein isoforms 1/2/3/4/5	3.13	2.20	2	1	1	1	727	77.3	7.83
13124784	Larval serum protein 1 gamma chain; AltName: Full=	191.31	45.34	1	46	49	105	772	93.3	5.44
**134467**	**Salivary glue protein Sgs-3; Flags: Precursor**	**3.96**	**18.57**	**1**	**2**	**2**	**3**	**307**	**32.2**	**9.89**
134470	Salivary glue protein Sgs-5; Flags: Precursor	18.89	41.10	1	6	6	10	163	18.8	7.72
135396	Tubulin alpha-1 chain	9.89	20.44	3	5	5	7	450	49.9	5.14
14286126	G protein alpha i subunit; AltName: Full=Guanine nu	0.00	2.54	1	1	1	1	355	40.6	7.64
1703117	Actin-57B; Flags: Precursor	20.98	31.65	4	2	8	11	376	41.8	5.39
1709616	Protein disulfide-isomerase; Short=PDI; Short=dPDI	2.11	5.04	1	2	2	2	496	55.7	4.82
1711388	Salivary glue protein Sgs-4; Flags: Precursor	21.64	6.40	1	2	2	8	297	32.3	8.91
17380352	Elongation factor 2; Short=EF-2	5.02	2.84	1	1	1	2	844	94.4	6.60
17380353	Protein crooked neck	0.00	1.57	1	1	1	1	702	84.2	5.94
209572654	Vesicle-fusing ATPase 2; AltName: Full=N-ethylma	5.59	7.31	2	4	4	4	752	83.4	6.74
224487970	Eukaryotic translation initiation factor 3 subunit C; S	1.92	1.21	1	1	1	2	910	105.6	6.06
2493029	ATP synthase subunit beta, mitochondrial; Flags: Pre	0.00	11.40	1	1	1	1	228	23.9	6.21
25089940	Conserved oligomeric Golgi complex subunit 7; Sho	1.97	1.62	1	1	1	1	742	84.0	5.85
25089946	Conserved oligomeric Golgi complex subunit 5; Sho	1.72	6.52	1	3	3	3	751	84.9	6.70
25091355	Tubulin beta-1 chain; AltName: Full=Beta-1-tubulin	6.16	19.02	2	3	3	7	447	50.1	4.86
266311	Heat shock 70 kDa protein cognate 4; AltName: Full	8.65	6.30	8	1	3	3	651	71.1	5.52
302595965	Polyubiquitin; Contains: RecName: Full=Ubiquitin;	4.37	45.87	3	3	3	4	763	85.7	7.66
30580507	Synapse-associated protein of 47 kDa	2.64	3.63	1	1	1	1	551	56.9	4.56
33302633	Probable phosphorylase b kinase regulatory subunit b	0.00	3.11	1	1	1	1	1093	124.8	6.81
33518622	Dipeptidyl peptidase 3; AltName: Full=Dipeptidyl ar	34.91	23.92	1	16	16	25	786	89.1	6.43
41018372	Unc-112-related protein; AltName: Full=Fermitin-1	3.08	2.12	1	1	1	1	708	80.4	7.72
41019481	Larval serum protein 1 alpha chain; AltName: Full=F	134.87	28.43	1	27	28	61	816	98.8	6.14
46397771	Histone H3	0.00	23.53	1	1	1	2	136	15.4	11.27
47606749	ATP synthase subunit beta, mitochondrial; Flags: Pre	0.00	3.17	1	1	1	1	505	54.1	5.27
50402125	Histone H4	4.33	21.36	1	2	2	2	103	11.4	11.36
50402136	Histone H2A	0.00	5.65	2	1	1	1	124	13.4	10.73
51315873	N-acetylgalactosaminyltransferase 6; AltName: Full=	4.91	3.90	1	2	2	2	666	76.9	6.25
51316103	Fragile X mental retardation syndrome-related protei	4.03	3.95	1	2	2	2	684	76.0	8.29
543797	Fructose-bisphosphate aldolase	3.22	3.88	1	1	1	1	361	39.0	7.40
55584057	Heat shock 70 kDa protein cognate 3; AltName: Full	12.26	8.84	7	2	4	4	656	72.2	5.36
68067863	Larval serum protein 2; Short=LSP-2; AltName: Full	10.29	4.99	1	4	4	4	701	83.3	6.35
68565081	Apolipophorins; AltName: Full=Retinoid- and fatty a	0.00	0.81	1	2	2	2	3351	372.4	7.97
73620969	ATP-dependent 6-phosphofructokinase; Short=ATP-	9.71	7.87	1	4	4	4	788	86.6	6.83
74861726	Coatomer subunit gamma; AltName: Full=Gamma-c	1.87	0.91	1	1	1	1	883	97.6	5.67
74869662	Cullin-associated NEDD8-dissociated protein 1; AltN	0.00	0.80	1	1	1	1	1248	139.3	5.87
74871183	Enhancer of mRNA-decapping protein 3	0.00	2.50	1	1	1	1	680	73.4	8.43
74871800	Poly(U)-specific endoribonuclease homolog; AltNam	6.37	3.38	1	2	2	3	592	63.6	8.88
74947986	ATP-dependent RNA helicase bel; AltName: Full=P	14.30	15.79	2	7	7	7	798	85.0	7.53
74948009	E3 UFM1-protein ligase 1 homolog	12.33	9.59	1	7	7	7	782	87.4	6.57
75029476	DNA replication licensing factor Mcm7; AltName: F	1.78	1.81	1	1	1	1	720	81.2	6.99
82592514	Protein no-on-transient A; AltName: Full=Puff-speci	2.11	6.00	1	3	3	3	700	76.9	9.35

Supplementary Table 3. Sequences of primers used throughout the manuscript.

Name	Sequence
pgant9A	Forward: 5'-GCGGAATTCGATATGGCCTTCATCTGGCGGCGAC-3'<br>Reverse: 5'-CGCTCTAGACTCAACTTCGAGCTGTCGTAG-3'
**pIB-*pgant9A* or *pgant9B* expression vector**	Forward: 5'-CGCACGCGTACACGGATGACACCCGGC GT-3'<br>Reverse: 5'-CTTGCGGCCGCTCACAACTTCGAGCTGTCGTAG-3'
***Sgs3*-∆**	Forward: 5'-TGGATGCCCCACCACACCTAAGCCGTG-3'<br>Reverse: 5'-GTGGTGGGGCATCCACAATCGCAACAGTTG-3'
Sgs3-T-Rich	Forward: 5'-CACAACACCCACCACACCTAAGCCGTG-3'<br>Reverse: 5'-GTGGTGGGTGTT GTGCACGGAAGTTGCG-3'
***pgant9* (C-terminal)**	Forward: 5'-CGACGACTCCTGTCTCGATT-3'<br>Reverse: 5'-GCTTGTGATTGTTGGTGTCG-3'
***UAS-pgant9A^{OE}* or *UAS-pgant9B^{OE}* expression vector**	Forward: 5'-GCGGAATTCGATATGGCCTTCATCTGGCGGCGAC-3'<br>Reverse: 5'-CTTGCGGCCGCTCACAACTTCGAGCTGTCGTAG-3'
***CG30463/pgant9* locus**	Forward: 5'-GTCATTCACAGGCTGAGAACAAC-3'<br>Reverse: 5'-CTTAAGCCCAGCTACTATCTCAGG-3'
pgant9A	Forward: 5'-ATGGTATCTGGACAACATT-3'<br>Reverse: 5'-CTTCTTCTGGTGCTTCTT-3'
pgant9B	Forward: 5'-AATGGTATCTGGACAACA-3'<br>Reverse: 5'-GGCACTCATAAATGGAAA-3'
18S rRNA	Forward: 5'-GGACACGCAAACTTCTCAACAGC-3'<br>Reverse: 5'-AATCTTCAGAGCCAATCCTTATCCC-3'

Supplementary Methods

PGANT9 antibody preparation and test. A recombinant protein fragment (PGANT9A 513-650aa) was expressed in E.coli then used for antibody production in rabbits (GenScript). To verify the specificity of antibody, PGANT9A-FLAG or PGANT9B-FLAG was expressed in S2R+ cells and the cell lysates were used for Western Blotting. Membranes were blocked and incubated with PGANT9 antibody (1:1000) or FLAG antibody (Sigma, 1:1000), and developed with IRDye 680LT-conjugated anti-rabbit IgG (Li-COR, 1:10,000) and IRDye 800LT conjugated anti-mouse IgG (Li-COR, 1:10,000) secondary antibody. Membranes were washed with PBST, and scanned using a Li-COR Odyssey Infrared Imaging System.
